# An Approach for Flux and Thickness Scaling of Cone Calorimeter Data for Predicting the Pyrolysis of Materials

**DOI:** 10.1007/s10694-025-01767-1

**Published:** 2025-06-12

**Authors:** Jason Floyd, Jonathan Hodges

**Affiliations:** 1 Fire Safety Research Institute, UL Research Institutes, Columbia, MD 21045 USA; 2 Research, Development, Testing and Evaluation Division, Jensen Hughes, Blacksburg, VA 24060 USA

**Keywords:** Pyrolysis, Computational fluid dynamics, Fire model, Fire growth

## Abstract

This paper presents an engineering approach to modeling pyrolysis. Instead of attempting to define detailed solid phase reactions, this method dynamically scales data from cone calorimeter tests of a material performed for different cone fluxes and/or sample thicknesses. The method can utilize data from multiple cone exposures and/or multiple sample thicknesses. The method compares the model predicted heat flux to a burning surface to the reference heat flux for a cone calorimeter test. The reference heat flux is the flame heat flux plus that portion of the cone heat flux that reaches the sample. In this method the reference heat flux is determined by table lookup of FDS simulations of a range of fuels and fuel burning rates in a cone geometry. The paper provides verification and validation of the approach. Validation is performed at multiple scales including a large series of 1D simulations for 141 materials, a cone calorimeter geometry for PMMA, a single burning item geometry for PMMA, a stack of wood pallets in a corner, and a room corner test at three length scales using plywood or fiber reinforced polymer. The method performs best for polymer and wood based materials. Specifically, it performed best when data from multiple cone exposures was self similar with a monotonic progression in peak burning rate as a function of exposure. The method showed a high degree of grid independence. The method also showed similar or improved performance to simulations using the detailed solid phase kinetics.

**Supplementary Information:**

The online version contains supplementary material available at 10.1007/s10694-025-01767-1.

## Introduction

When modeling the impact of a fire, the size and location of the fire over time is a critical model input. This can be done as a prescribed fire or a predicted fire. For a prescribed fire, the spatial and temporal details of the fire are specified by the modeler. This approach is typical in performance-based design (PBD) for fire safety applications or when performing a fire risk assessment. A prescribed fire may be a single item whose heat release rate has been measured or a constructed fire consisting of a growth, steady-burning, and decay phase. In either case, the model inputs are the heat release rate over time and the location and surface area of the fire. For predicted fires, the modeler instead provides inputs that allow the model to predict the fire size, surface area, and location over time.

The heat release rate (HRR) of a burning fuel depends on the complex interactions of condensed phase pyrolysis, gas phase combustion, and heat transfer back to the surface. Each of these processes are themselves complex set of physical phenomena. Focusing on solely the condensed phase, modeling its pyrolysis involves modeling of heat transfer to and in the condensed phase, condensed phase chemical reactions, potentially the transport of pyrolyzates within the condensed phase, and diffusion of gases from the exposed surface into the condensed phase. To do this from first principles requires a significant amount of data related to material properties and material reactions. While the fire safety community has made significant progress in quantifying material and reaction properties in recent years [1], it is still a process that requires a great deal of effort, knowledge, and specialized equipment. Three general approaches to predicting fire growth and spread have been used in fire safety science.

The first and simplest approach is defining an ignitable object in the model. The heat flux to or surface temperature of the object is monitored. When a predefined ignition point is reached, the object ignites and burns following a predefined heat release rate for the entire object. While this approach is simple to implement, a major challenge is locating burning rate data for the exact object being modeled. There are databases of single burning items which fire modelers can use as a reference, such as the SFPE handbook [2], the Fire Calorimetery Database [3], the Burning Item Database [4], the RISE Fire Database [5], and the EPRI-NRC transient combustibles database [6]. A significant limitation of this method is single object data is often obtained by burning the object in the open under a hood calorimeter. Heat feedback enhancement (e.g., hot layer) or reduction (e.g., vitiation) due to an enclosure cannot be accounted for nor can the effect of changes in the physical location and method of ignition. In addition, while these experiments provide valuable data points, the specific fuel package in a design basis often varies from available experiments (e.g., different materials, combustible mass, etc.). In a design application, engineering judgment must be used to assess the applicability of these individual tests and how to scale measured data to the specific application.

The second general approach replaces full object data with the time dependent pyrolysis rate of a material based on bench-scale test data such as the cone calorimeter in ISO 5660 [7]. With this approach, heat transfer to an object made of the material is modeled. Different regions of the object (for example a single grid cell in a computational fluid dynamics (CFD) model) are allowed to ignite when reaching a predefined ignition temperature. At ignition, the region then follows the measured pyrolysis rate per unit area from the bench-scale test. With this approach fire can spread across an object rather than having the entire object ignite and burn. An advantage of this approach is bench-scale testing is relatively inexpensive compared to full object testing. A limitation is that while fire can spread over an object with this method, each region still burns based on the bench-scale data without changes in the burning rate if heat feedback differs from the exposure used in testing. Since this approach requires predicting surface temperature, it does require material properties for heat transfer. However, since it applies bench-scale data post-ignition, the burning rate is not coupled with the surface temperature post ignition.

The final approach seeks to describe the small-scale processes and transport phenomena occurring within the fuel. This is typically based on an Arrhenius kinetics formulation [1]:

1 $$\begin{aligned} r_{i}=A_{i}exp\left( -\frac{E_{i}}{RT}\right) \zeta _{1}\zeta _{2}...\zeta _{N} \end{aligned}$$

where $r_{i}$ is the reaction rate, $A_{i}$ and $E_{i}$ are the Arrhenius pre-exponential factor and activation energy of the *i*-th reaction, *T* is the temperature, *R* is the molar gas constant, and $\zeta _{j}$ is the impact of the *j*-th material component’s concentration on $r_{i}$. A key advantage of this approach is the the material decomposition rate will change based on the predicted thermal exposure to the material. The challenge is determining the number of Arrhenius reactions and their parameters along with the properties required to determine the in-depth temperature profile within the material. This approach also fully couples the reaction rate to the solid temperature and errors in material properties, Arrhenius reaction data, and the predicted heat feedback can combine mutiplicatively. While optimization approaches exist to determine these properties [8, 9], the overall effort needed to quantify these properties may not be feasible for specific fire safety applications. This approach can be challenging to apply outside of research applications of fire models.

The third approach is currently technically and logistically challenging for widespread commercial application. The second approach cannot adapt itself to actual conditions being predicted in a model. There is great appeal in being able to use cone calorimeter data. It is relatively quick and inexpensive to run a small number of cone tests for a material. These observations led to the development of an enhanced second approach called the scaling-based pyrolysis model (Spyro). This approach was developed to bridge the gap between the second and third modeling approaches [10]. The first version of Spyro dynamically scaled the HRR of a material measured at one exposure in a cone calorimeter experiment to the exposures predicted in a CFD fire model (such as Fire Dynamics Simulator (FDS)). The authors demonstrated the potential of the model by examining the statistical performance of the model for a large set of materials available in the public literature.

While the first version of the Spyro model showed promise, there were several limitations in its initial development, three of which are addressed in this work. The original formulation was not able to take advantage of the full set of data collected when material testing in a cone calorimeter is done at multiple exposures (as is typical). As a result, the model was always operating in an extrapolation mode when in many cases it should be possible to interpolate between different cone exposures. Second, the original model used a fixed reference heat flux in scaling which assumed the flame heat flux occurring during the experiment was constant. This flux, which is not a measured parameter during a cone test, had to be specified by the user. Lastly, the model was limited to materials which are the same thickness in the model as tested in the cone calorimeter experiment. This assumes that the total energy released per unit area does not vary between the bench-scale and installed configurations.

This paper presents an enhanced Spyro model that can use cone data sets for a material consisting of different material thicknesses and/or multiple exposures. The enhanced model has been implemented in the open-source CFD fire model, Fire Dynamics Simulator (FDS) and is available for use by the community as of the release of FDS 6.10.0 (commit used in this work was Version 9f96527).

## Methodology

The method developed in this research adapts the original Spyro model [10] to incorporate the use of multiple cone calorimeter exposures plus the effects of varying initial thickness on the predicted HRR of the material. The method is based on interpolation and scaling of the burning rate curves based on non-dimensional analysis of heat transfer during cone calorimeter testing. In addition, an empirical formulation for the time-varying reference heat flux is developed based on CFD predicted values and integrated into the method.

### Original Spyro Model

The original Spyro model used an effective heat of gasification approach to scale the measured burning rate from a single cone calorimeter experiment to the heat flux predicted by a fire model, e.g. FDS. The effective heat of gasification approach starts with a steady burning rate, ${\dot{m}}''$ in a material:

2 $$\begin{aligned} {\dot{q}}''_{net}={\dot{q}}''_{ref} -{\dot{q}}'''_{store} \Delta x - {\dot{q}}''_{cond}={\dot{m}}'' \Delta H_g \end{aligned}$$

where ${{\dot{q}}}''_{net}$ is the net heat flux into a surface in kW/$\hbox {m}^2$ and $\Delta H_g$ is the heat of gasification in kJ/kg. The net flux in Eq. 2 includes the net convective and radiative flux to the surface (${\dot{q}}''_{ref}$), energy used to raise the material temperature (${\dot{q}}'''_{store} \Delta x$), and the heat flux conducted into the material (${\dot{q}}''_{cond}$). If a quasi-steady burning rate is considered, where the temperature of the surface is not changing, then the energy storage part becomes zero leaving:

3 $$\begin{aligned} {\dot{q}}''_{net}={\dot{q}}''_{ref} - {\dot{q}}''_{cond} \end{aligned}$$

For a cone test, ${\dot{q}}''_{ref}$ is given by:

4 $$\begin{aligned} {\dot{q}}''_{ref}={\dot{q}}''_{flame} + {\dot{q}}''_{cone} (1-\Gamma ) \end{aligned}$$

where ${\dot{q}}''_{flame}$ is the flame heat flux in kW/$\hbox {m}^2$, ${\dot{q}}''_{cone}$ is the pre-flame cone exposure flux in kW/$\hbox {m}^2$, and $\Gamma$ is the fraction of that flux absorbed by the flame. Since most solid combustible materials are insulative in nature, once a material is burning, ${\dot{q}}''_{cond}$ is generally small compared to the incident flux. Ignoring it gives

5 $$\begin{aligned} {\dot{q}}''_{ref} \approx {\dot{m}}'' \Delta H_g \end{aligned}$$

Assuming that $\Delta H_g$ is invariant with exposure, then for the same material exposed to two different values of ${\dot{q}}''_{ref}$ results in:

6 $$\begin{aligned} \frac{{\dot{m}}''_2}{{\dot{m}}''_1} = \frac{{\dot{q}}''_{ref,2}}{{\dot{q}}''_{ref,1}} \end{aligned}$$

or

7 $$\begin{aligned} {\dot{m}}''_2 = {\dot{m}}''_1 \frac{{\dot{q}}''_{ref,2}}{{\dot{q}}''_{ref,1}} \end{aligned}$$

That is, ${\dot{m}}''$ scales directly with the change in ${\dot{q}}''_{ref}$. If the same mass of material was exposed to two different ${\dot{q}}''_{ref}$, then in order to preserve the total ${\dot{m}}''$ when scaling the burning rate, time would also need to be scaled. Mass consumed is simply the burning rate times the burning duration, $\delta t$ in s. Therefore, then $\delta t$ scales inversely with ${\dot{m}}''$:

8 $$\begin{aligned} \delta t_2 = \delta t_1 \frac{{\dot{q}}''_{ref,1}}{{\dot{q}}''_{ref,2}} \end{aligned}$$

The original formulation of Spyro used Eqs. 7 and  8 directly to scale the exposure, with a fixed ${\dot{q}}''_{flame}$ and $\Gamma =0$. The value of ${\dot{q}}''_{flame}$ was an input the user had to specify.

Improving the original approach to allow for multiple fluxes and/or multiple thicknesses plus reduce user effects due to ${\dot{q}}''_{ref}$ required developing a method to have FDS determine ${\dot{q}}''_{ref}$ plus methodologies for scaling based on flux and thickness. These are covered in the three subsections that follow.

### Determining the Reference Heat Flux

A key step in the methodology is establishing ${\dot{q}}''_{ref}(t)$ for each set of cone test data (combination of exposure and thickness) being provided as input to the simulation. While the cone flux, ${\dot{q}}''_{cone}$, is known, neither ${\dot{q}}''_{flame}$ nor $\Gamma$ have known values over the duration of the cone test. While there have been some experimental efforts to measure ${\dot{q}}''_{ref}$, those efforts are limited to a small number of materials, they include the combined effect of ${\dot{q}}''_{flame}$ and $\Gamma$, and the measurements have some degree of intrusiveness on the test itself.

The original Spyro method considered simple hand type calculations of the view factor from a burning cone sample along with a small number of FDS simulations. This was used to estimate an average ${\dot{q}}''_{ref}$ which was used as an input parameter. However, in actuality this parameter is a function of time and ideally a time-dependent ${\dot{q}}''_{ref}$ should be generated within FDS based on the cone test data and material combustion properties.

**Fig. 1:**
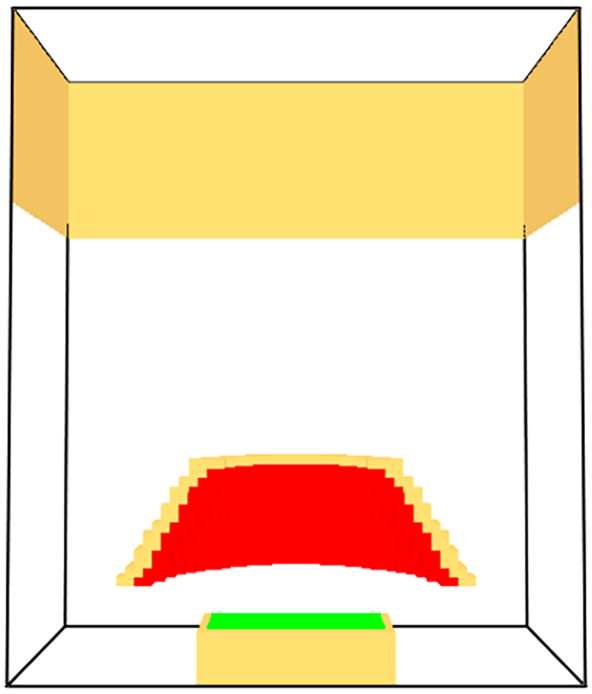
Geometry used to determine flame heat flux and and flame absorption of cone radiation. Red is the cone heater, and green is the material sample

Rather than attempt to define a hand calculation based approach for determining ${\dot{q}}''_{ref}$, it was instead determined via modeling with FDS 6.9.1. A cone calorimeter geometry was defined with a total domain extent of 0.32 m by 0.32 m by 0.38 m and a uniform grid resolution of 5 mm putting 20 cells across the sample. The main geometric features were the sample holder with a vent defining the sample and the cone heater. The cone was set to a fixed temperature of 792.07 °C which resulted in an average incident heat flux of 50 kW/$\hbox {m}^2$ to the full sample with range of 49 to 51 kW/$\hbox {m}^2$ over a radius of 5 cm from the sample center. This met the ASTM 1354 [11] requirement for ±2% over the central 5 cm. The sample surface was set to a fixed temperature of 300 °C (intended as a representative pyrolysis temperature) and a specified burning rate. The geometry is shown in Figure 1. All exterior boundaries were set as open except for the upper walls, which were solid to represent the cone hood.

Physically, ${\dot{q}}''_{flame}$ and $\Gamma$ are related to plume dynamics (e.g., air entrainment) and the absorption and emission of radiation. The impact of the user-defined combustion reaction at variable fire sizes was investigated through a parametric study. Simulations were performed for all 180 permutations of five fuel heats of combustion,$\Delta H_c$ , (10, 20, 30, 40, and 50 MJ/kg), five soot yields, $Y_s$, (0, 1, 2, 5, 10, and 20%) with CO yield set equal to the soot yield, and six radiative fractions, $\chi _r$, (10, 20, 30, 40, 50, and 60%). The ranges were selected to encompass most fuels typically found in the built environment. Each simulation consisted of three sequential phases: the burner stepping through a range of heat release rate per unit area (HRRPUA) with the cone off (100, 200, 400, 800, 1200, 1600, 2000, 2500, and 3000 kW/$\hbox {m}^2$), the burner stepping through the same set of HRRPUA with the cone on, and the burner off with the sample at ambient temperature and the cone on. Each step was 20 s long and the heat flux to the sample was averaged over the last 15 s for each step. In the first phase, ${\dot{q}}''_{flame}$ is determined as the average flux over the sample. The second phase determines $(1-\Gamma ){\dot{q}}''_{cone}$ as the excess incident flux over ${\dot{q}}''_{flame}$ from the first phase. Since ${\dot{q}}''_{cone}$ a constant, the third phase determines $\Gamma$. An example input file is provided in the supplementary material.

The gas phase fuel chemistry inputs for FDS were defined to maintain a constant oxygen heat of combustion (EPUMO2) of 13,100 kJ/kg. For the 50 MJ/kg fuel a methane like fuel was assumed ranging from $\hbox {CH}_{3.3}$ for 0% soot yield to $\hbox {CH}_{6.2}$ for 20% soot yield. For the 40 MJ/g fuel a $\hbox {CH}_2$ like fuel was assumed (i.e., a longer chain hydrocarbon normalized to one carbon atom) ranging from $\hbox {CH}_{0.95}$ for 0% soot yield to $\hbox {CH}_{2.9}$ for 20% soot yield. For the remaining heats of combustion a cellulosic like fuel ($\hbox {CH}_2$ $\hbox {O}_x$) was assumed with *x* ranging from 0.30 for 30 MJ/kg and 0% soot yield to 0.73 for 10 MJ/kg and 20% soot yield.

**Fig. 2:**
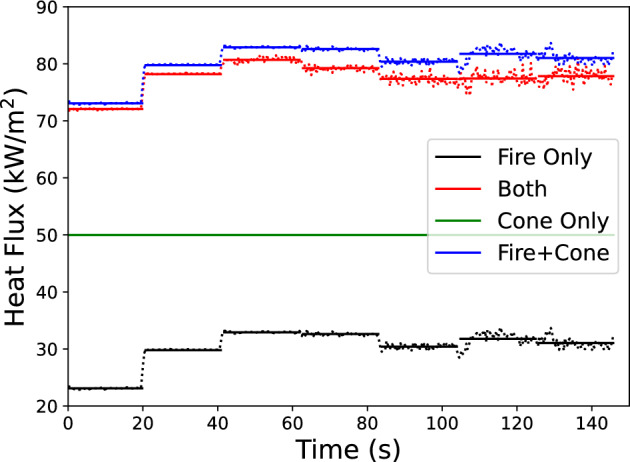
Results of the cone simulation for a 40 MJ/kg fuel with $Y_s$=5% and a $\chi _r$=30%. Dotted lines are simulation results and solid lines are the averages over each HRRPUA (100 to 3000 kW/$\hbox {m}^{2}$ from left to right)

An exemplar result is shown in Figure 2 which is the results for the 40 MJ/kg fuel with $Y_s$=5% and $\chi _r$=30%. The black Fire Only curve is ${\dot{q}}''_{flame}$, the red Both curve is ${\dot{q}}''_{ref}$ which is ${\dot{q}}''_{flame}+(1-\Gamma ){\dot{q}}''_{cone}$, the green Cone only curve is ${\dot{q}}''_{cone}$, and the blue Fire+Cone is ${\dot{q}}''_{flame}+{\dot{q}}''_{cone}$. The value of $\Gamma$ is given by the relative difference between the blue and red curves as compared to the blue curve. In essence the end result of this process is a calibration of the expected FDS prediction for a cone calorimeter test, and it is assumed that this calibration holds for larger samples of material with changing orientation.

**Fig. 3:**
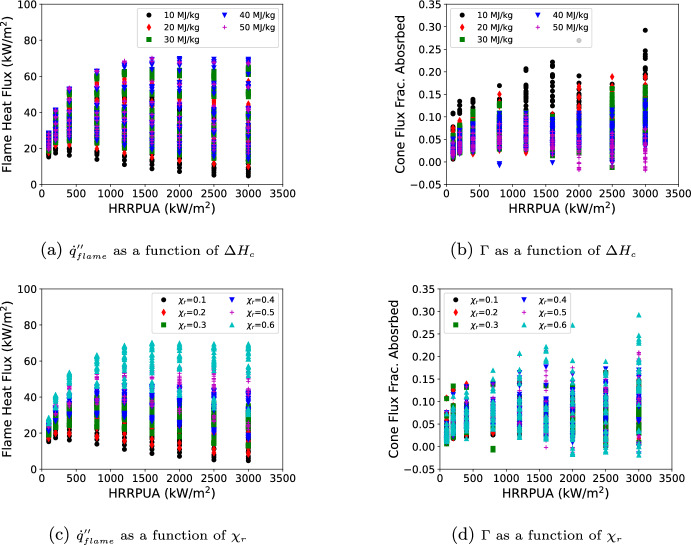
Results of simulations performed to determine ${\dot{q}}''_{ref}$

A summary of all results is shown in Figure 3 with ${\dot{q}}''_{flame}$ and $\Gamma$ plotted against HRRPUA and colored by either $\Delta H_c$ or $\Gamma$. A few observations are made on the data presented. The ${\dot{q}}''_{flame}$ generally increased with the HRRPUA up until 1600 kW/$\hbox {m}^2$ at which point ${\dot{q}}''_{flame}$ plateaued. The ${\dot{q}}''_{flame}$ showed a increasing trend with increasing $\Delta H_c$. This was a result of lower $\Delta H_c$ requiring more fuel mass flow for the same HRRPUA. There was, unsurprisingly, a clear trend of increasing ${\dot{q}}''_{flame}$ with increasing $\chi _r$. The value for $\Gamma$ showed a decreasing trend with increasing $\Delta H_c$; however, no clear trend was seen for $\Gamma$ and $\chi _r$.

There is limited measured data on heat fluxes in cone calorimeter tests. While multiple researchers have reported data, it covers a narrow range of materials. Fluxes have been measured with water cooled gauges which replace combustible material with an inert gauge that is also cold. Additionally, determination of flame heat fluxes has been by subtraction of the cone exposure without accounting for the flame absorption of the cone. Typically fluxes have been measured at the center of a cone sample; however, one effort did measure near the edge. This makes direct comparison with the flame heat flux values in this work challenging as they were taken as the average flux over the sample which was at 300 °C rather than close to ambient. Some of the available data includes McCoy et al. [12], Leventon et al. [13], Li et al. [14], Hermouet et al. [15], and Beaulieu and Dempsey [16]. McCoy measured ${\dot{q}}''_{flame}$ values for three polymer materials: POM, PMMA, and HIPS. Leventon measured the flame heat flux in a vertical wall where the ignition source was removed post-ignition to multiple polymers including: ABS, HIPS, PMMA, POM, and PP. These vertical fluxes are not directly comparable to a cone test; however, the Spyro method assumes that similar flame fluxes would result given the same fuel and burning rate. These were taken as being similar to edge fluxes when tabulated. Beaulieu measured the center flux for POM and PMMA but the work does not clearly report the cone exposure for the reported value. Li measured the center to MDF. Hermouet measured the center flux to ABS.

Table 1 compares the measured values at the cone sample center and edge with the average ${\dot{q}}''_{flame}$ from modeling. Heats of combustion and radiative fraction were taken from the referenced tests. Soot yields were taken from the SFPE Handbook [17]. Measured ${\dot{q}}''_{flame}$ for cone tests were adjusted using the FDS predicted $\Gamma$. In general the average predicted FDS values lie between the center and edge values for the materials when both values exist.

**Table 1:** Comparison of FDS predicted ${\dot{q}}''_{flame}$ to measured values [12–16]

Plastic	$\Delta H_c$	$\chi _r$	$Y_s$	Center	Edge	${\dot{q}}''_{flame}$
	MJ/kg	%	%	kW/$\hbox {m}^2$	kW/$\hbox {m}^2$	kW/$\hbox {m}^2$
ABS	36.4	46	10	22–23	43–48	34–35
HIPS	30.0–39.2	50	20	26–28	45–53	32–50
MDF	12	22–27	2.0	20	–	23–26
PMMA	24.5–25.5	33	1.5	17–18	33–35	33–34
POM	15.3–15.9	20	10	16	23–41	22–23
PP	41.0	48	1	–	37–45	29–32

The set of ${\dot{q}}''_{flame}$ and $\Gamma$ were tabulated as two four dimensional arrays of dimension (5,31,21,6). The array elements were ($\Delta H_c$ in increments of 10 MJ/kg, $Y_s$ in increments of 1%, HRRPUA in increments of 100 kW/$\hbox {m}^2$, $\chi _r$ in increments of 1%). A python script performed multi-dimensional, linear interpolation of the the FDS results to populate the array. The two sets of interpolated results for ${\dot{q}}''_{flame}$ and $\Gamma$ were then output as Fortran code that was added to the FDS source. In FDS, the reference flux is determined by calling a function with $\Delta H_c$, $Y_s$, HRRPUA, $\chi _r$, and ${\dot{q}}''_{cone}$. This function linearly interpolates the stored arrays over all four dimensions of the input variables to determine ${\dot{q}}''_{flame}$ and $\Gamma$. Then Eq. 4 is used to determine ${\dot{q}}''_{ref}$.

If the cone test data is from an inerted test apparatus, then the function for ${\dot{q}}''_{ref}$ simply returns the cone exposure without any correction for absorption. This assumes that the small quantities of pyrolzates (diluted by the inerting coflow) between the sample and the cone, do not significantly absorb any flux from the cone.

### Scaling with Data from Multiple Exposures

**Fig. 4:**
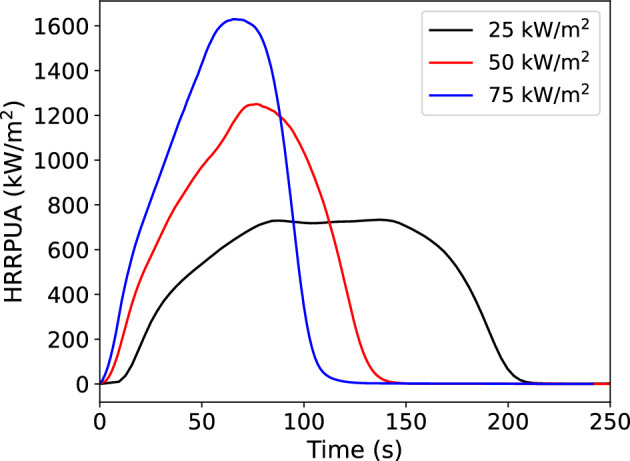
Plot of cone calorimeter data for black PMMA

Consider a set of cone data for a material as shown in Fig. 4. This data is for black PMMA and is taken from the Fire Safety Research Institute’s (FSRI) Materials and Products Database [18] (data is available at https://materials.fsri.org). If this data is to be interpolated in time based on a model prediction, the current model time needs to be correlated to test time for each of the cone curves. For example, if one was predicting the HRRPUA for a 35 kW/$\hbox {m}^2$ test, at the time of peak HRRPUA at 35 kW/$\hbox {m}^2$, the interpolation model should be using the burning rates at 87 s on the 25 kW/$\hbox {m}^2$ curve and 77 s on the 50 kW/$\hbox {m}^2$ curve.

Since $\Delta H_c$ is assumed invariant with exposure, if Eq. 5 is integrated over time for each cone exposure, then the result is an equivalence of the sample mass consumed at a time and the integrated reference flux at that time. This equivalence can be generated for each cone exposure. During a simulation, once a grid cell ignites, the predicted reference flux is integrated over time. The equivalence between integrated flux and mass consumed then lets the model determine the equivalent time for each cone test’s burning rate curve. If the Spyro model were a perfect representation, then this would mean that plotting the integrated burning rate over time against the integrated ${\dot{q}}''_{ref}$ for different cone exposures would result in a single collapsed curve. This concept was observed in Lyon’s 2024 work assessing the combustible potential of materials in which he noted similar behavior for materials when plotted in this manner [19].

**Fig. 5:**
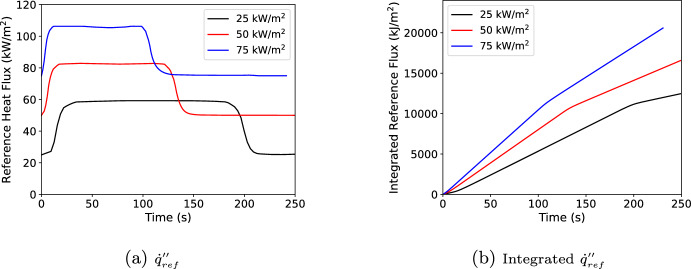
${\dot{q}}''_{ref}$ and integrated ${\dot{q}}''_{ref}$ for black PMMA

Using the PMMA cone curves, $\Delta H_c$=24.9 MJ/kg, $Y_s$=1.8%  [20] with the default FDS $\chi _r$=0.35, and the data created in Section 2.2, ${\dot{q}}''_{ref}$ and its integral over time can be constructed for each exposure as shown in Fig. 5. In FDS, the integrated ${\dot{q}}''_{ref}$ is stored as energy vs. time rather than time vs. energy.

Continuing with the prior example of predicting a 35 kW/$\hbox {m}^2$ test, assume that a wall cell has reached a point where it has a current incident flux of 75 kW/$\hbox {m}^2$, a current integrated incident flux of 6000 kJ/$\hbox {m}^2$, and the the FDS time step is 10 s (a large number for FDS but picked for the purpose of the example). For the current time step the updated integrated incident flux would 6450 kJ/$\hbox {m}^2$. Using the integrated ${\dot{q}}''_{ref}$ curves in Figure 5b this would be times of 119 and 81 s for the 25 kW/$\hbox {m}^2$ and 50 kW/$\hbox {m}^2$ tests. These times correspond to HRRPUA values of 725 and 1237 kW/$\hbox {m}^2$ with reference fluxes of 59 and 82 kW/$\hbox {m}^2$. Interpolating to 75 kW/$\hbox {m}^2$ would give a burning rate of 1074 kW/$\hbox {m}^2$. Interpolating straight to the HRRPUA curve is the simplest approach; however, it could mean missing local peaks, local minimums, or other trends in the HRRPUA data over the time step. Instead, the cone HRRPUA curves are also integrated in time. The beginning of time step times at 6000 kJ/$\hbox {m}^2$ are 111 and 75 s. If instead of getting the HRRPUA at the effective times, the integrated HRRPUA is used, then the 25 kW/$\hbox {m}^2$ curve releases 5500 kJ/$\hbox {m}^2$ over the effective time step and the 50 kW/$\hbox {m}^2$ curve releases 6800 kJ/$\hbox {m}^2$ over the effective time step of 8 s. This gives average HRRPUA values of 723 and 1245 kJ/$\hbox {m}^2$ which interpolates to 1079 kJ/$\hbox {m}^2$. This is larger than just using the HRRPUA curve. Looking at the HRRPUA curve it can be seen that it is increasing and decreasing over the effective times respectively for 25 kW/$\hbox {m}^2$ and 50 kW/$\hbox {m}^2$. Using the integrated HRRPUA curve captures that net effect, preserving the correct total sample mass loss.

### Scaling Material Thickness

Heat transfer into solid materials can be characterized by two primary non-dimensional groups. These are the Fourier, $\textrm{Fo}$, and Biot, $\textrm{Bi}$, numbers. $\textrm{Fo}$ is the ratio of the heat conduction rate to the rate of thermal energy storage in a solid, and can be thought of as a dimensionless time [21]. It is defined as

9 $$\begin{aligned} \textrm{Fo}=\frac{\alpha }{\delta ^{2}}t \end{aligned}$$

where $\alpha$ is the thermal diffusivity, $\delta$ is the the thickness of the solid, and *t* is time. $\textrm{Bi}$ is the ratio of the internal thermal resistance of a solid to the external resistance of the surface boundary layer [21]:

10 $$\begin{aligned} \textrm{Bi}=\frac{h \delta }{k} \end{aligned}$$

where *h* is the heat transfer coefficient, and *k* is the thermal conductivity of the solid. This can be considered as a dimensionless thickness. A small Biot number ($\le$0.1) indicates a thermally-thin material capable of being treated as a lumped mass [21]. The traditional formulation of $\textrm{Bi}$ assumes that external exposure of the solid is related primarily to surface convection. However, this assumption is not valid in the case of burning solid fuels where there is also a significant radiative exposure, ${\dot{q}}_{r}''$, from the flame and in the case of a cone calorimeter test, ${\dot{q}}''_{cone}(1-\Gamma )$. An alternative form of $\textrm{Bi}$ is proposed in this work which combines the effect of surface convection and radiation in the external resistance term:

11 $$\begin{aligned} \mathrm {Bi^{*}}=\frac{h_{c}+h_{r}}{k/\delta } \end{aligned}$$

where *h* has been split into two components for convection, $h_{c}$, and radiation, $h_{r}$. The radiation heat transfer coefficient is calculated based on the linearized formulation [21]:

12 $$\begin{aligned} h_{r}=\varepsilon \sigma \left( T_{r}+T_{s}\right) \left( T_{r}^{2}+T_{s}^{2}\right) \end{aligned}$$

where $\varepsilon$ is the surface emissivity, $\sigma$ is the Stefan-Boltzmann constant, $T_{r}$ is the radiative temperature, and $T_{s}$ is the surface temperature. The radiative temperature is an effective temperature representative of the energy absorbed at the surface assuming a view factor of 1.

The $\textrm{Fo}$ and $\mathrm {Bi^*}$ numbers can be combined to create a new dimensionless number: $\mathrm {FoBi^*}$:

13 $$\begin{aligned} \mathrm {FoBi^{*}} = \frac{h}{\rho c \delta } t \end{aligned}$$

With this one can scale time for the same material with different thicknesses by taking the ratio of their $\mathrm {FoBi^*}$ numbers.

14 $$\begin{aligned} t_2 = \frac{h_1 \delta _2}{h_2 \delta _1} t_1 \end{aligned}$$

If materials of two thicknesses are both burning during a cone test of the same exposure, then the *h* values will be similar leading to

15 $$\begin{aligned} \frac{t_2}{t_1} \approx \frac{\delta _2}{\delta _1} \end{aligned}$$

When providing FDS inputs for thickness scaling, two sets of thicknesses are required. The first is the thickness of the material as it is being modeled in FDS. For FDS users, this is the normal THICKNESS input on SURF. The second is the thickness associated with each cone test. This second thickness should be taken as the nominal thickness of the tested sample. For example, one has tested plywood sold as 1 cm and 1.5 cm thicknesses at exposures of 25, 50, and 75 kW/$\hbox {m}^2$. To do this one would acquire a sheet of each thickness and cut it into samples for the cone tests. Each sample would have an actual thickness that is slightly different from the manufacturer’s labeled thickness. The 1 cm sheet might have had samples that were 0.95, 1.02, and 1.03 cm thick. When inputting the tested thickness, these should all be given the same 1 cm thickness. This way if the two sheets were each tested at three fluxes, one would input six sets of cone data grouped into two thickness rather than six sets of cone data grouped into six thicknesses.

### Algorithm for Multiple Thickness+Flux Scaling

Below is the algorithm used by FDS to scale the burning rate based on a set of cone calorimeter data containing multiple cone exposures for multiple sample thicknesses.

1. Preprocessing:
  - Compute the time scaling factor $s=\delta _{sim}/\delta _{cone}$ for each thickness. It is important to note that for Spyro, the sample thickness is the nominal material thickness. For example, one is testing a nominally 1 cm thick piece of material. A large piece of the material is obtained and cut into 10 cm by 10 cm samples for testing. Measuring the thickness of each individual sample will result in some range of thicknesses near 1 cm perhaps ranging from 0.95 to 1.06 cm. As inputs to FDS, these should all be considered as being 1 cm.
  - Integrate each cone test’s HRRPUA curve over time.
  - For each cone test, use that test’s HRRPUA curve and cone exposure along with the fuel heat of combustion, soot yield, and radiative fraction to compute the curve for ${\dot{q}}''_{ref}$.
  - Integrate each ${\dot{q}}''_{ref}$ over time to get $E_{ref}(t)$, the integrated reference flux as a function of time.
  - Invert each $E_{ref}(t)$ from the prior step to get $t_{ref}(E_{ref})$, the reference time associated with a specific integrated integrated reference flux.
2. During simulation after a cell reaches its ignition temperature:
  - For each thickness, compute the integrated incident flux using the simulation time step scaled by *s* and the simulation predicted incident flux.
  - Get the scaled reference time, $t_{ref,i}$, for each HRRPUA curve, *i*, using the inverted curves from Step 1e.
  - Interpolate the integrated HRRPUA curves from Step 1b using the corresponding $t_{ref,i}$.
  - Using the difference from the prior time step integrated HRRPUA value and value for $t_{ref,i}$ (both are zero for the first time step after ignition), compute the average HRRPUA for each cone curve provided as input. If $t_{ref,i}$ for a curve is beyond the final value for that curve, use that curve’s final HRRPUA value scaled with the ratio of the current incident flux to ${\dot{q}}''_{ref,i}(t_{ref,i})$.
  - For each thickness interpolate the HRRPUA value from the prior step using the current incident flux the associated ${\dot{q}}''_{ref,i}(t_{ref,i})$. If the FDS incident heat flux is above or below the range of ${\dot{q}}''_{ref,i}$ values for that thickness, then simply apply Eq. 7.
  - Interpolate the results of the prior step using the simulated thickness and tested thicknesses. If the simulated thickness is above or below the range of tested thicknesses, then simply use the determined burning rate for the smallest or largest thickness as appropriate. The time scaling from Step 2b already accounts for the thickness scale per Eq. 8.
  - Store the current integrated HRRPUA and $t_{ref,i}$ for each curve for use in the next time step.
  - Repeat from Step 2a for each new simulation time step.

### Example

**Fig. 6:**
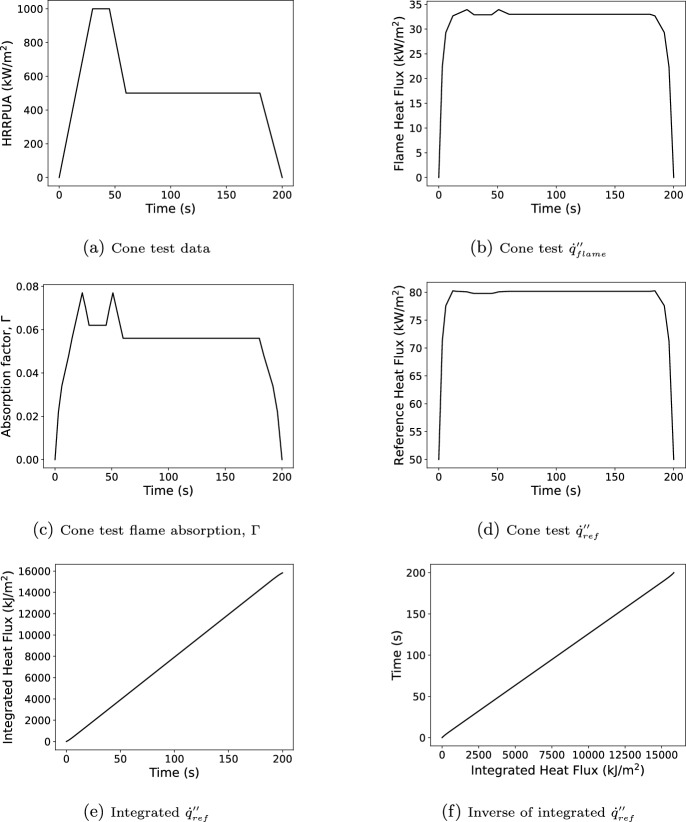
Sequence of Spyro pre-processing steps being applied to data created to be representative of a 50 kW/$\hbox {m}^2$ standard cone test

This section provides an example of the scaling process using created data representing a single cone test. For this example, the material has $\Delta H_c=30$ MJ/kg, $Y_s=5$ %, and $\chi _r=0.30$ and was tested in a cone calorimeter at 50 kW/$\hbox {m}^2$. Figure 6 shows the HRRPUA from the cone test (created data) along with the corresponding ${\dot{q}}''_{flame}$, $\Gamma$, and ${\dot{q}}''_{ref}$ as determined from the interpolation tables generated in Section 2.2. Also shown in the figure are the time integral of ${\dot{q}}''_{ref}$ and its inverse. This corresponds to Step 1 in Section 2.5 and is done during processing of the input file.

**Fig. 7:**
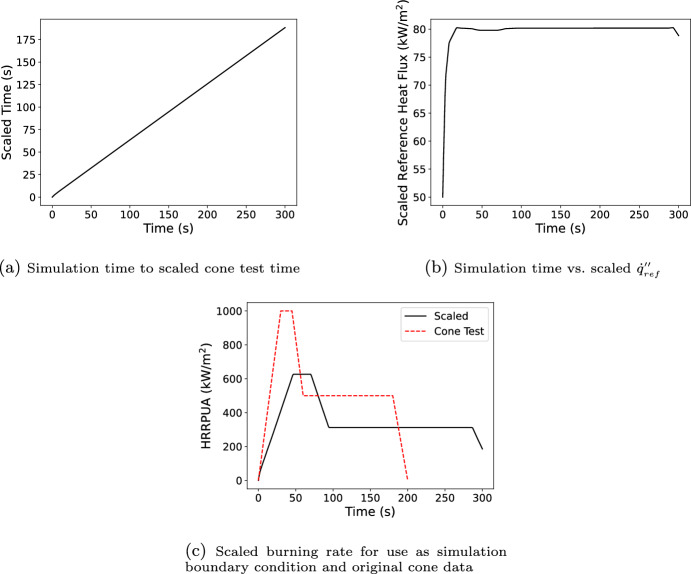
Sequence of Spyro in-simulation steps for the example of scaling created data representative of a 50 kW/$\hbox {m}^2$ standard cone test for a model of a 50 kW/$\hbox {m}^2$ inert cone test

Next assume the material is being modeled in an inert cone with the same exposure 50 kW/$\hbox {m}^2$. As an inert cone model, the simulation predicted ${\dot{q}}''_{ref}$ would be 50 kW/$\hbox {m}^2$, and the integral of the predicted ${\dot{q}}''_{ref}$ at time *t* would be 50*t* kJ/$\hbox {m}^2$ (i.e., Step 1c in Section 2.5). With no ${\dot{q}}''_{flame}$ and $\Gamma$ effectively zero, the peak mass loss rate of the material is expected to be lower and the decomposition to occur for longer than the for the cone test which was not inerted. Figure 7 shows the result of converting the modeled flux integral to the scaled test time using the inverse curve (Figure 6f), the test ${\dot{q}}''_{ref}$ at the scaled time, and the scaled burning rate to be used in the simulation which was obtained by scaling the measured burning rate (Figure 6a) at the scaled time by the ratio of the predicted ${\dot{q}}''_{ref}$ at model time *t* to the measured ${\dot{q}}''_{ref}$ at the scaled time (Figure 6b). This represents Step 2a through Step 2e in Section 2.5.

## Verification

### Flux Scaling

**Fig. 8:**
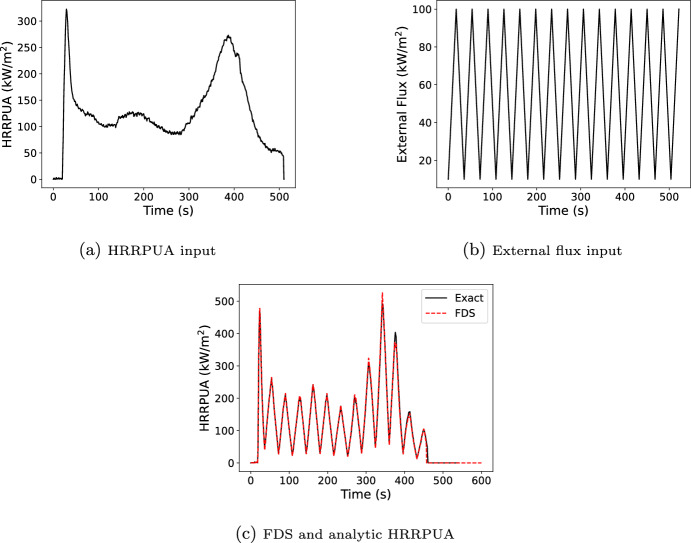
Inputs and results for the flux scaling verification case

In this verification example, data from a single, inert cone test at 50 kW/$\hbox {m}^2$ (Figure 8a) was exposed to a time varying external flux (Figure 8b). The surface ignition temperature was set to 0 K so that the surface immediately begins to use the Spyro algorithm. A python script was written for the algorithm described in Section 2.5 and used for comparison with FDS predictions (Figure 8c).

### Flux and Thickness Scaling

**Fig. 9:**
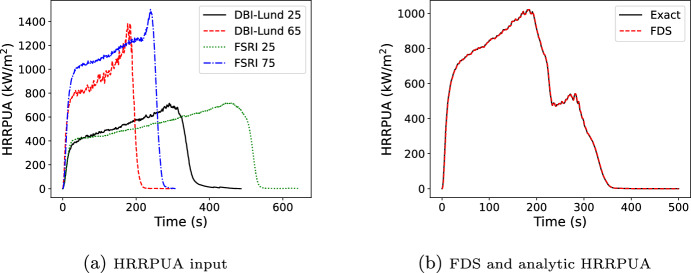
Inputs and results for the flux and thickness scaling verification case

In this verification example a 7.3 mm sample of material is exposed to a 50 kW/$\hbox {m}^2$ external flux. Data is provided for the same material at a thickness of 6.2 mm at 25 and 65 kW/$\hbox {m}^2$ and a thickness of 8.4 mm at 25 and 75 kW/$\hbox {m}^2$, see Figure 9a (note this is the DBI-Lund and FSRI PMMA data used in Section 4.2). The input data is treated as inert cone data. As with the prior verification case, the surface ignition temperature was set to 0 K. The expected results predicted with the python script are used for comparison with FDS predictions, see Figure 9b. FDS predicts the expected HRRPUA.

## Validation

Validation of the new scaling approach was done using both 1D and 3D geometries. The 1D cases were solid phase only calculations that simply looked to reproduce cone calorimeter data at one flux or thickness using test data from different fluxes or thicknesses. The 3D cases included a 3D cone, a single burning item (SBI) test of black PMMA, tests of stacks of wood pallets in a corner, and room corner lining tests at three length scales for two materials. For the cone, SBI, and wood pallet cases simulations using kinetics for pyrolysis were performed in addition to the scaling approach. For those simulations FDS inputs were identical in terms of geometry and boundary conditions except for those inputs specifically related to pyrolysis (e.g., kinetics parameters or cone data).

### 1D Solid Phase

#### Multiple-Flux scaling

The multiple-flux scaling approach was validated against 141 materials from publicly available datasets of cone test data where the same material had flaming ignition at multiple cone exposures. These include 2 tests from the Aalto wood experiments [22], 8 tests of polymers from the US Federal Aviation Administration (FAA) [23, 24], 8 wood materials from the US Forest Products Labs (FPL) [25], 67 materials from the Fire Safety Research Institute’s (FSRI) materials database [18], 15 materials from Jensen Hughes (JH) [26, 27], and 41 materials from the Research Institutes of Sweden (RISE) [5]. Materials were divided into categories of polymers, wood-based (wood and wood based products), mixtures (multi-layered, polymers with fibers, etc.), and other (shingles, rubbers, etc.). See the original Spyro publication for a description of these datasets [10]. The additional materials added in this work were from new commits to the FSRI material database since the original Spyro publication. Minimal review was done for the data obtained. Materials or individual cone tests were removed if ignition did not occur since the Spyro method is only applied post-ignition. Experience from MacFP-2 [28] showed there can be significant lab-to-lab variance in the result of cone tests. However, for this Section and the next, the goal is validation of the overall scaling method. Each 1D simulation only uses data from a single entity. If bias or errors in process exist, presumably those apply to all data collected by that entity for that material and should not impact the overall conclusions when scaling the data.

Every tested exposure was modeled in FDS using the cone data from other available exposures (i.e., if test data existed at 25, 50, and 75 kW/$\hbox {m}^2$, the 50 kW/$\hbox {m}^2$ model used only the 25 and 75 kW/$\hbox {m}^2$ data). This resulted in 516 total comparisons over the 141 sets of material data. These simulations were done as solid phase only simulations. When running in solid phase only mode, FDS uses the current predicted burning rate as input to the empirical approach for determining ${\dot{q}}''_{ref}$ to obtain the predicted value for the method. For materials where there was no reported $Y_s$ a value of 5% was used. As the goal of this exercise was to simply validate the scaling approach and not the ability of FDS to predict material heating to ignition, predefined ignition times based on the experimental values were specified in the FDS input files (later validation exercises will look at the entire process). All the input files are available from the FDS GitHub repository in the Scaling_Pyrolysis folder under Validation. A summary of the materials including thickness, category, test lab, and tested fluxes used is provided as supplementary material.

Validation looked at the peak HRRPUA and peak time for the first peak in the HRRPUA, the peak HRRPUA and peak time for the largest remaining peak, and the duration of time over which the total heat released represented 10% to 90% of the energy release. If there was only one clearly defined peak, then that dataset was dropped when evaluated the remaining peak statistics. For some materials the duration of one cone exposure result in a significantly larger or smaller total heat release than the other exposures. In those cases the duration of heat release was limited to the smallest total heat release of the curves used for a specific scaled flux. For example, if the lowest exposure had a very low total heat release but the middle and high exposure were similar, then comparisons for the low and middle would be based on the low total heat release but the high would not since it only uses the middle for scaling. Validation is expressed in terms of two statistical parameters: standard deviation $\sigma$, and bias, $\delta$). In this section and the next parameters were obtained following the process detailed in Section 4.4 of the FDS Validation Guide [29].

**Fig. 10:**
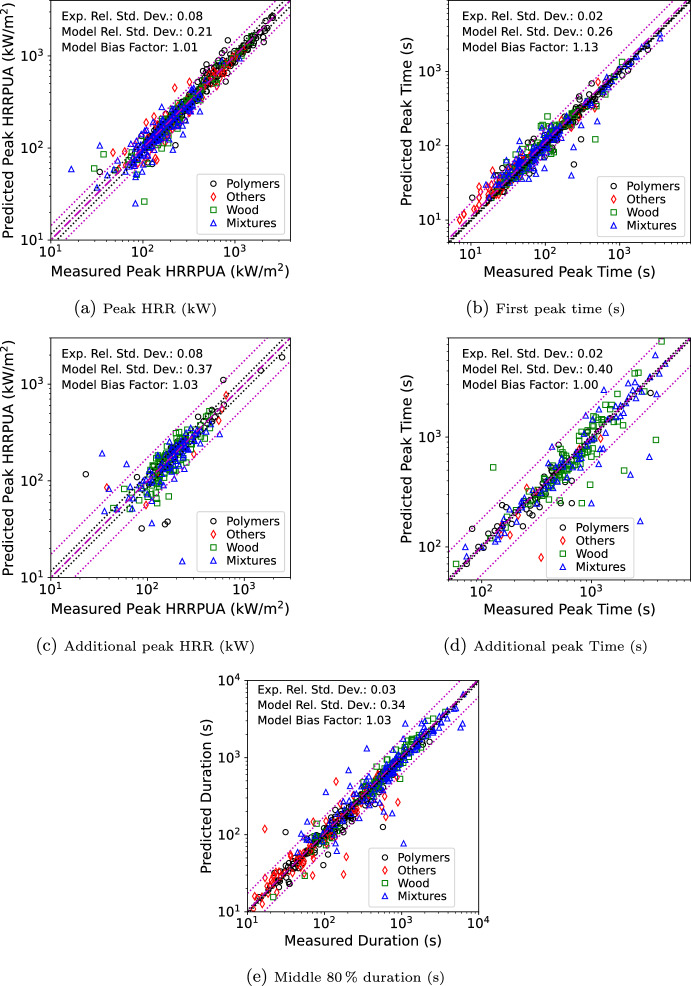
Scatterplots of measured vs. FDS predicted values for multiple-flux scaling

Results of the validation cases for the peak burning rate are shown in Figure 10 for the first peak, the largest additional peak, and the middle 80% burning duration. Results are colored by material type. In the figure the dash-dot line is the mean (prediction=measurement) and dotted lines are two standard deviations ($2 \sigma$) from the mean with black for the experiment and magenta for the FDS predictions where the mean is corrected for bias ($\delta$). Results are shown in Table 2 for all items and by category where the error is reported as one standard deviation ($1 \sigma )$. By group, polymers perform the best followed by wood-based, other, and mixtures. Statistics are better for the first peak magnitude and time than for the remaining peaks. The initial peak was unbiased for magnitude but biased late in peak time. The next largest peak was generally unbiased in time but slightly over predicted in magnitude.

**Table 2:** Flux scaling validation results

	First Peak				Additional Peak				Duration	
	HRRPUA		Time		HRRPUA		Time		Middle 80%	
Category	$\sigma$	$\delta$	$\sigma$	$\delta$	$\sigma$	$\delta$	$\sigma$	$\delta$	$\sigma$	$\delta$
All	0.21	1.01	0.32	1.16	0.36	1.03	0.45	1.02	0.34	1.03
Polymers	0.15	1.02	0.33	1.14	0.40	1.09	0.33	1.06	0.24	0.98
Others	0.22	1.01	0.20	1.20	0.37	1.06	0.48	0.92	0.48	1.07
Wood-Based	0.21	0.99	0.29	1.11	0.25	0.98	0.37	1.01	0.19	1.01
Mixtures	0.28	1.00	0.37	1.19	0.46	1.05	0.58	1.03	0.44	1.10

**Fig. 11:**
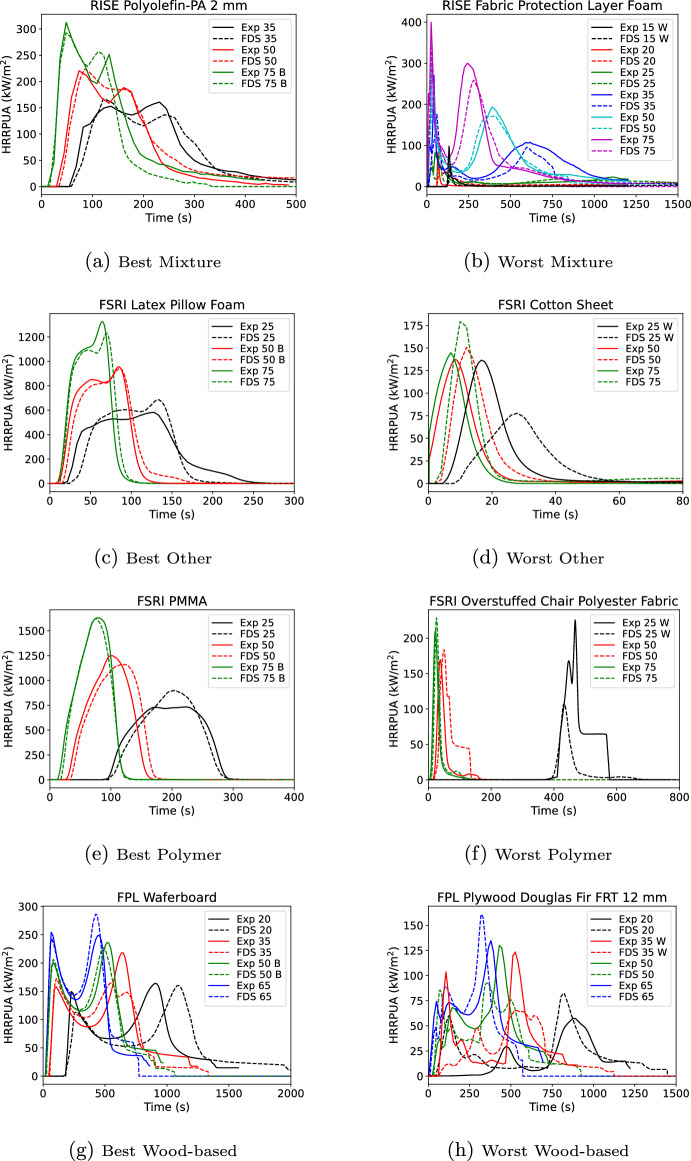
Best (left) and worst (right) material per category for flux scaling

Figure 11 shows the material containing the smallest and largest overall error taken as the square root of the sum of the variance for the first peak HRRPUA, first peak time, and duration for releasing 80% of the total heat release rate. Additional peak data was not included as many materials had only a single peak. Errors were evaluated at each exposure. The specific exposure that is the best or worst is indicated respectively with a B or W in the legend.

For the best mixture, self similar curve shapes are seen of two overlapping peaks followed by a decay. The curves are very self similar. For the worst mixture, the 35 through 75 kW/$\hbox {m}^2$ data showed a well defined first peak post-ignition that rapidly drops before starting a second peak. At lower fluxes there was only a single peak and the sample appeared to self-extinguish. The higher exposures were well predicted, but the Spyro method does not work well when samples do not have sustained ignition. The best other material shows a self similar curve shape which rises quickly to a plateau which is followed by a peak prior to decay. For the worst other, the material was a thin cotton sheet. The material ignited quickly with slightly longer ignition times (0–5 s) as the exposure decreases. Once ignited all the materials achieved the same peak HRRPUA over similar burning durations. As a result at the lowest exposure of 25 kW/$\hbox {m}^2$, the Spyro method flattened out the 50 kW/$\hbox {m}^2$ which was not seen in the data. The best polymer was PMMA. Similar to the best other material, the curves were very self similar. The worst polymer was another thin material, the fabric covering for a chair cushion. Similar to the cotton sheet, once ignited each exposure burned with similar peak HRRPUA values which resulted in under predicting the 25 kW/$\hbox {m}^2$ curve when scaling the 50 kW/$\hbox {m}^2$ data. The best wood material followed the pattern seen in other material types, the curves bounding the exposure being predicted were self similar. For the worst wood material, an FR plywood, the ignition time at 25 kW/$\hbox {m}^2$ was an order of magnitude longer than at 35 kW/$\hbox {m}^2$ with a first peak that was 25% of the peak at 35 kW/$\hbox {m}^2$. With such a dissimilar presentation, Spyro would not be expected to perform well.

Some general observations on Spyro were derived from this exercise. Very thin materials may be challenging for Spyro; however, in actual usage performance would be better than indicated here. If one was predicting a cotton sheet using all three exposures, then the scaling error at or near 25 kW/$\hbox {m}^2$ would not have occurred as one would have used the 25 kW/$\hbox {m}^2$ data. Use of cone data where the material sees brief or intermittent ignition may be a challenge for Spyro. In this case even using all data might not lend to good performance at lower fluxes. For example, in the worst mixture, sustained burning occurs somewhere between 25 and 35 kW/$\hbox {m}^2$. For some exposures below 35 kW/$\hbox {m}^2$ it would be likely that sustained burning would still happen, but post first peak the scaling method would average in the near zero burning at 25 kW/$\hbox {m}^2$. Performance was best when curves were self similar with similar areas under the curves. When using Spyro in practice, performing a similar exercise of predicting exposures that were tested would provide a good indication if the Spyro method was suitable for a particular materials.

#### Multiple-Thickness Scaling

The multiple-thickness scaling approach was validated using a subset of the FAA polymer data and RISE data. Five polymers (polycarbonate (PC), polyvinyl chloride (PVC), polymethyl methacrylate (PMMA), high density polyethylene (HDPE), and high impact polystyrene (HIPS)) in the FAA database were tested at multiple fluxes and multiple thicknesses. Eight materials in the RISE database were tested at multiple fluxes (FR particle board, polyolefin/EPR, polyolefin/PA, polyolefin/XLPE, PVC/PVC, PVC/XLPE, RPVC/XLPE, and zhpolylefin/XLPE). Each combination where multiple thicknesses were tested at the same exposure were simulated in a manner similar to the flux scaling validation (i.e., if tested at the same exposure with thicknesses of 3, 6, and 9 mm, the 6 mm model used only the 3 and 9 mm data). This resulted in 105 total comparisons. Using only datasets of the same flux was done to keep the scope of this validation exercise to just the thickness scaling approach. An example input file is provided in the supplementary material (this was a modified version of the flux scaling input from the prior section).

Statistics were generated in the same manner as for the flux scaling in the prior section; however, there were no other materials and the single wood material lacked clear multiple peaks during scaling.

Table 3 provides a summary of the validation results. The overall results were worse than for flux scaling; however, this was not a true reflection. The single wood material was an FR plywood which the flux scaling showed was a particularly challenging material. With only a small number of materials for thickness scaling this extreme outlier skewed the statistics. Additionally, there was a higher proportion of mixtures which had a higher error than the polymers. The polymer only and mixture only results were similar to those determined for flux scaling. Removing the wood outlier did improve the overall statistics, especially for the first peak size and the duration.

**Table 3:** Thickness scaling validation results

	First peak				Additional peak				Duration	
	HRRPUA		Time		HRRPUA		Time		Middle 80%	
Category	$\sigma$	$\delta$	$\sigma$	$\delta$	$\sigma$	$\delta$	$\sigma$	$\delta$	$\sigma$	$\delta$
All	0.37	1.08	0.91	1.76	0.33	1.02	0.63	1.01	0.54	1.27
Polymers	0.17	1.03	0.33	1.07	0.26	1.07	0.42	0.89	0.34	1.13
Wood-Based	0.95	1.43	0.81	1.65	N/A	N/A	N/A	N/A	1.53	5.60
Mixtures	0.39	1.11	1.27	3.04	0.95	1.43	0.81	1.65	0.44	1.16
All No Wood	0.29	1.07	0.92	1.78	0.33	1.02	0.62	1.01	0.39	1.14

**Fig. 12:**
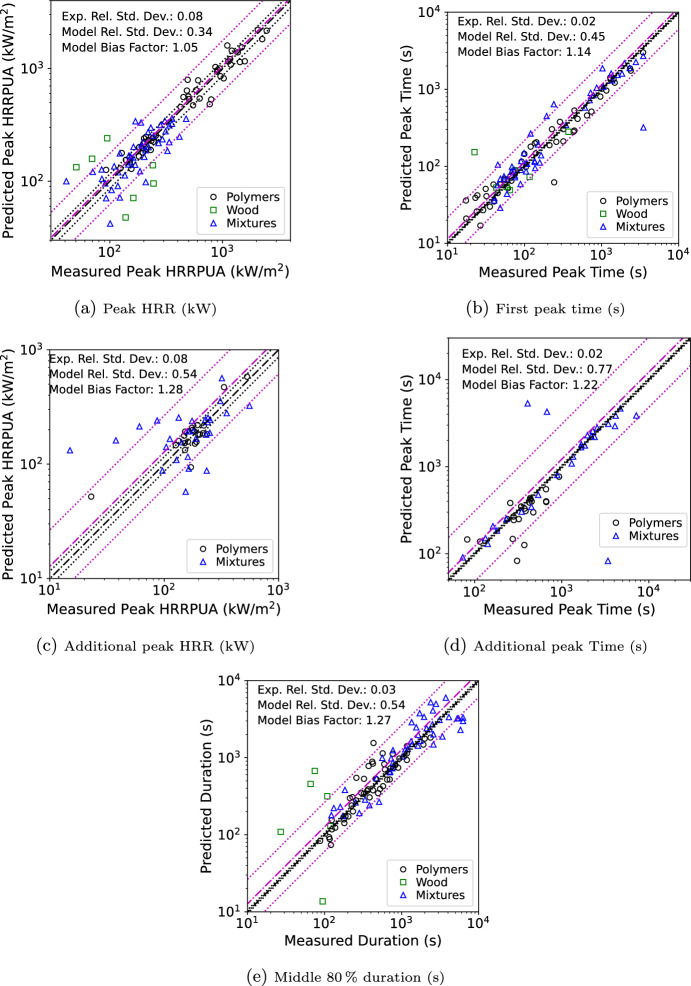
Scatterplots of measured vs. FDS predicted values for multiple-thickness scaling

Figure 12 shows the first peak results, the additional peak results, and the middle 80% burning duration results.

**Fig. 13:**
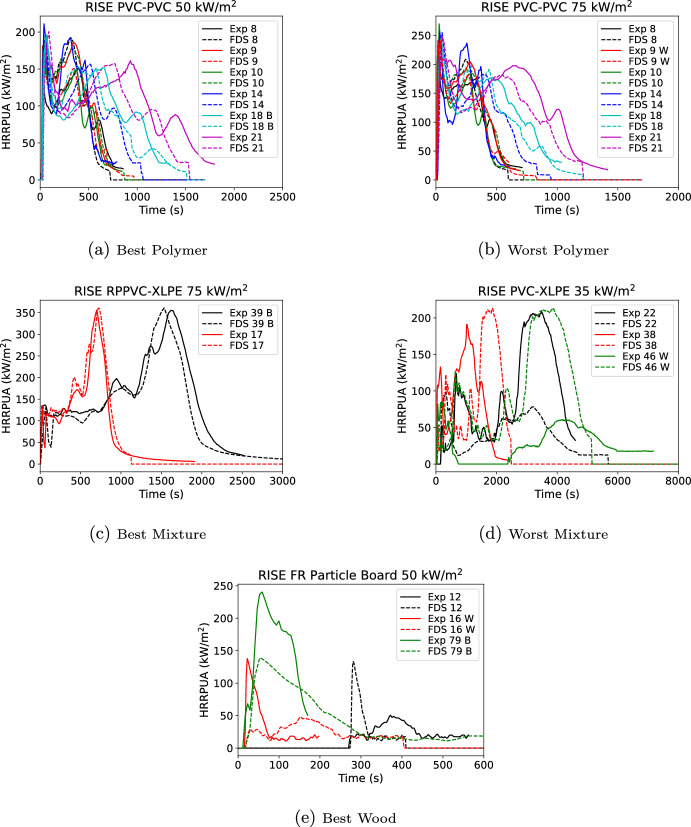
Best (left) and worst (right) first peak HRRPUA (kW/$\hbox {m}^2$) per category for thickness scaling

The best (B) and worst (W) material based on peak HRRPUA for polymers, mixtures, and wood are shown in Fig. 13. The figure shows the material containing the smallest and largest overall error taken as the square root of the sum of the variance for the first peak HRRPUA, first peak time, and duration for releasing 80% of the total heat release rate. Additional peak data was not included as many materials had only a single peak. Errors were evaluated at each thickness and exposure.

Interestingly, the best and worst polymer were two different thicknesses of PVC-PVC. At first glance the various worst curves look reasonable. The driving factor in the error was the first peak time of the 75 kW/$\hbox {m}^2$ curve was twice the experimental time leading to a high overall error metric even though peak height had a 1% error and duration had a 6% error. However, the magnitude of the time error was only 18 s out of an approximately 600 s total burning duration. For the best mixture, it was again a case with very self similar curves. For the worst mixture, it was again a case where the worst exposure at this thickness did not have sustained burning leading to errors when scaling from the thinner thickness at the same exposure which did. For wood there was only one material with two thicknesses. Neither thickness were predicted well, and the best and worst single combination of flux and thickness both occurred at the 50 kW/$\hbox {m}^2$ exposure. The wood was an FR material similar to the worst wood in the flux scaling. The data from the three thicknesses at 50 kW/$\hbox {m}^2$ do not have self similar shapes and show behavior without a clear trend with increasing thickness.

### PMMA Tests

The Measurement & Computation of Fire Phenomena (MaCFP) working group of the International Association of Fire Safety Science created a combined experimental and modeling test series to evaluate developing kinetics schemes for solid phase pyrolysis and then applying those schemes to model parallel panel and single burning item (SBI) tests. Black PMMA was selected as the solid material. Samples were sent to various laboratories for testing and development of a kinetic scheme for pyrolysis. Details on the testing, bulk material properties (density, specific heat, and conductivity), and solid phase kinetic schemes can be found at https://github.com/MaCFP/matl-db/tree/master/PMMA.

This section modeled two geometries using the Spyro method and detailed kinetics. The Spyro method was used for three sets of cone data: a 50 kW/$\hbox {m}^2$ inert gasification test performed by NIST (no flame); a set of standard cone tests performed by DBI-Lund at exposures of 25, 50, and 65 kW/$\hbox {m}^2$; and a set of standard cone tests performed by FSRI (USA) at exposures of 25, 50, and 75 kW/$\hbox {m}^2$ for a slightly thicker PMMA sample (8.4 mm vs. 6.1 mm for the NIST and DBI-Lund tests). The detailed kinetics was performed using the 2021 UMD and 2023 DBI-Lund kinetics schemes. The cone and kinetic data was taken directly from the MaCFP GitHub repository given above. For both geometries the Spyro and detailed kinetics input files were identical in terms of geometry and domain boundary conditions. Only the solid phase inputs for pyrolysis rate differed. For the Spyro simulations, the bulk material properties (density, specific heat, and conductivity) from the UMD kinetics scheme were used. For the Spyro model, a scoping 1D simulation was run with the bulk material properties and the reported ignition times from the cone data. The surface temperature at ignition from the scoping simulation was used as the IGNITION_TEMPERATURE for the Spyro method.

#### Inert Cone (Gasification) and Standard Cone Test

In this validation exercise, FDS predictions were compared against two sets of tests. One test was a standard cone test performed by a joint effort of DBI (Denmark) and Lund University (Sweden), and the other was an inert gasification test ($\hbox {N}_2$ inerted) performed by NIST. The NIST test apparatus was essentially a standard cone inside of an small enclosure that was inerted with $\hbox {N}_2$. In the remainder of the paper this test apparatus is called the “inert cone”. Both tests used a 50 kW/$\hbox {m}^2$ exposure. FDS simulations used the same geometry model as used for developing the reference flux (Section 2.2). Example input files for both the kinetics approach and the Spyro approach are provided in the supplementary material. Simulations were done using the UMD and DBI-Lund kinetic schemes and using Spyro with cone data collected either by DBI-LUND (25, 50, and 65 kW/$\hbox {m}^2$) or FSRI (25, 50, and 75 kW/$\hbox {m}^2$). Spyro simulations were performed for either all three fluxes of cone data collected by the institution or each flux individually. The simulation using all of the DBI-LUND data was also run using the DBI-LUND bulk material properties with the IGNITION_TEMPERATURE re-calibrated to the new properties.

**Fig. 14:**
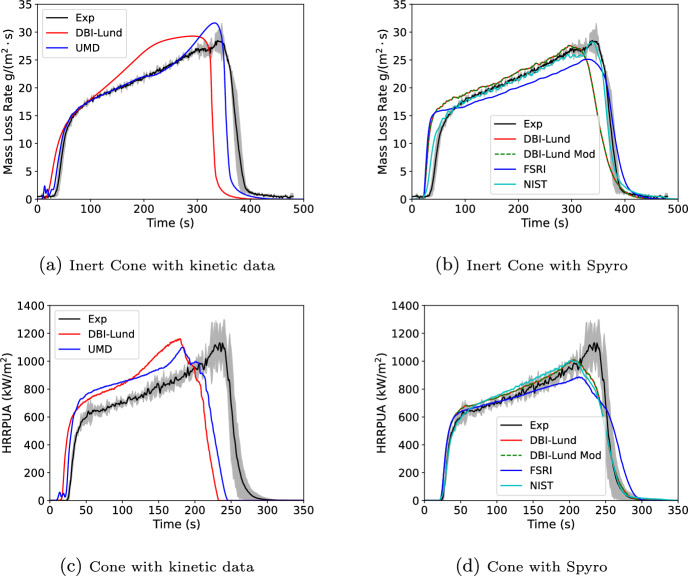
Inert (top) and non-Inert (bottom) cone test at 50 kW/$\hbox {m}^2$ compared to FDS predictions using detailed kinetics (left) and Spyro (right)

Results are shown in Figure 14. Some portion of the error should be attributed to the imposed flux. A cone type apparatus does not create a perfectly uniform incident flux on the sample, and that non-uniformity differed between the experiment and the model. This would result in some minor differences on the ignition and burnout time of any specific location on the sample. For both the inert and non-inert cone, the Spyro method more closely reproduces the measured data over all datasets when compared with the kinetics schemes. Additionally, the Spyro results show generally less spread in results when compared with the detailed kinetics approaches. For the inert cone data, the NIST Spyro simulation matches very closely to the measured data. If that FDS cone model perfectly represented the NIST cone this match should be exact. With the differences being minor, this indicates the FDS cone model reasonably reproduced the flux of the NIST inert gasification device. Using Spyro with inert test data, non-inert test data, or data from a minor change in thickness does not significantly impact the results. The same was true for using 1, 2, or 3 exposures (DBI-LUND results); however, as seen in Figure 4 the cone calorimeter curves for PMMA are very self similar making this material particularly amenable to the Spyro approach.

#### PMMA Single Burning Item Test

**Fig. 15:**
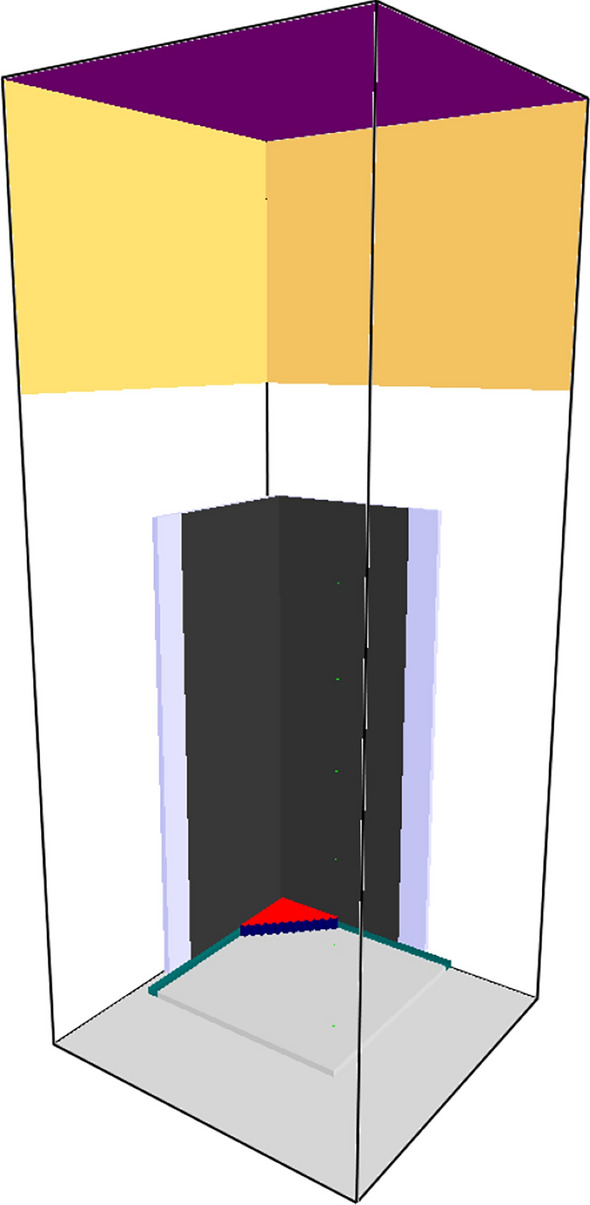
FDS SBI geometry. Red is propane burner, purple is hood exhaust, dark gray is PMMA, and light blue is Marinite fiberboard

In this validation exercise, FDS predictions are compared against a single burning item (SBI) test of black PMMA performed by the University of Maryland [20] under a hood calorimeter. A brief test summary and the experimental data are available at https://github.com/MaCFP/macfp-db/tree/master/Fire_Growth/UMD_SBI. Predictions were made using the two kinetic schemes and three sets of cone data used in the prior section including simulations using each of the three FSRI and DBI cone exposures alone. The 0.58 cm thick PMMA panels were 50 cm wide and 146 cm tall and were mounted on 1.27 cm thick Marinite boards that were 60 cm wide. A 30 kW propane sand burner (25 cm side length, right isosceles triangle) was used to ignite the PMMA. Simulations were run at 2 and 4 cm grid resolutions. The domain included the hood. FDS control functions were used to program the same calorimetry equation as used to process the test data. This allowed FDS to mimic the effect of smoke collection in the hood on the time dependence of the computed heat release rate plus account for the impact of the specific calorimetry equation used. Example input files for both the kinetics approach and the Spyro approach for the 4 cm grid resolution are provided in the supplementary material. Figure 15 shows the FDS geometry.

**Fig. 16:**
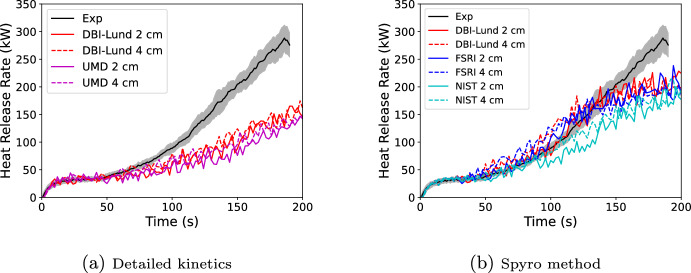
Comparison of FDS predicted SBI burning rate for black PMMA using detailed kinetics (left) or Spyro (right)

**Fig. 17:**
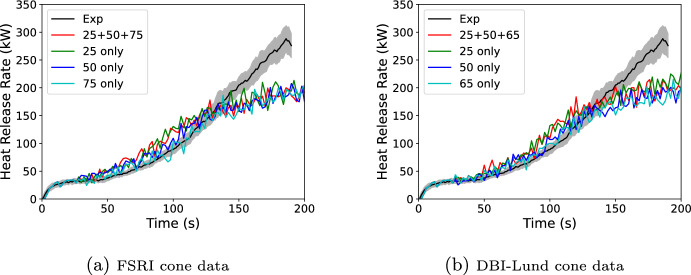
Comparison of FDS predicted SBI burning rate for black PMMA varying the cone data used for FSRI data (left) or DBI data (right)

Results for the kinetic schemes and for simulations with all cone exposures are shown in Figure 16. Results using each of the DBI-Lund and FSRI cone exposures alone are shown in Figure 17. The drop in the experimental HRR at test end was due to suppressing the fire to avoid overwhelming the hood calorimeter. Both the detailed kinetics and the Spyro methods show relatively little grid dependence at the two modeled resolutions. Both kinetics methods result in approximately one-half of the peak measured HRR at the suppression time. The three Spyro methods are approximately one-third low. The two Spyro simulations using a standard cone (DBI-Lund and FSRI) have similar predictions. The NIST data from an inert cone predicts approximately 10% lower values of the HRR than the two datasets from a standard cone. However, the prediction is still an improvement over the detailed kinetics. Performance was the same for three cone exposures vs. a single exposure; however, this was not surprising given the earlier 1D and 3D cone results for PMMA.

### Stacked Wood Pallets

On October 25, 2005 an instructor at a firefighter training facility died during a training evolution while adding new wood pallets to the burning remnants of pallets from prior training evolutions [30]. NIST was invited to assist in the post-incident investigation. As part of their activities, NIST performed a series of tests to measure the heat release rate of wood pallets stacked in similar configurations to those used in the training facility [31]. Two tests (tests 4 and 5) with similar stacking approaches and similar fuel masses to those at the training facility were selected for comparison with FDS using the Spyro model. The pallets were stacked in a room corner consisting of two 2.4 m by 2.4 m walls with a ceiling made with all surfaces made of gypsum wallboard. The room corner was placed under a hood calorimeter.

Pyrosim [32] was used to define a notional wood pallet representing the construction and size of pallets used in the NIST tests. Six copies of the pallets were rotated and stacked to mimic the stacking approach used in tests 4 and 5 which both used 6 pallets with a total mass of 97.8 kg. The pallets were defined with three surface types: a 1.9 cm thick wood surface for the tops and bottoms of the planks, a 3.8 cm thick wood surface used for the vertical faces of the stringers, and a 10 cm thick wood surface used for all other faces. The geometry was exported at 3.8 cm and 7.6 cm resolution. This meant that the voxelization differed between the two cases (i.e., different numbers and sizes of OBST inputs are generated at the different grid resolutions). The 7.6 cm geometry export was run with both 3.8 cm and 7.6 cm grids to investigate the impact of grid resolution on the same voxelization.

**Fig. 18:**
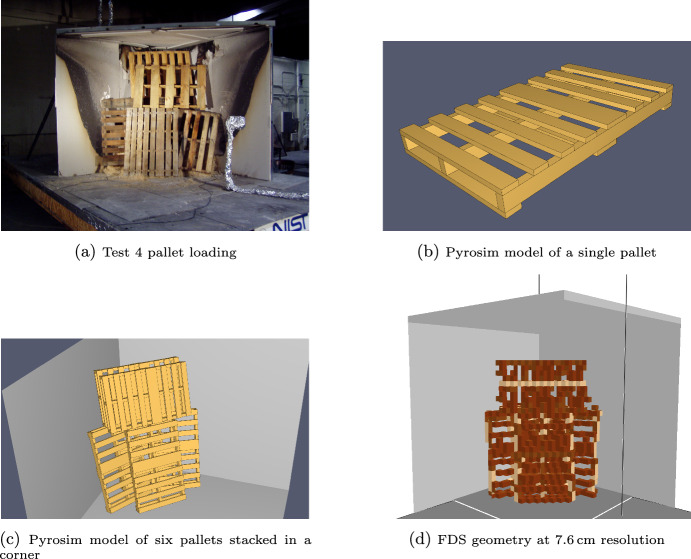
Geometry model development for open corner pallet stack

Figure 18 shows a pre-test photo of test 4, the Pyrosim pallet, the Pyrosim pallet stack, and the FDS geometry (7.6 cm). In the FDS geometry model, the room corner was extended 1.2 m from the ends of the corner and 1.2 m above the ceiling to contain the flames from the fire.

In the model, each pallet was assigned a mass of 16.3 kg (in testing pallet masses for each test ranged from 11 to 24.5 kg). Based on typical construction of three stringers, three planks on the pallet bottom, and a fully decked pallet top; the bottom planks, stringers, and top planks of each pallet were assigned respective masses of 2.7, 6.3, and 7.2 kg. The FDS obstructions were output from Pyrosim with labels indicating which pallet and which pallet component (top deck, stringer, bottom) they belonged to. The total volume of each component for each pallet was determined and used with the component mass to set the BULK_DENSITY input for the obstructions (with BULK_DENSITY set, FDS will remove an obstruction grid cell-by-grid cell when the pyrolyzed mass matches the mass given by BULK_DENSITY and the obstruction volume). The input file for the 7.6 cm grid is provided in the supplementary material.

Cone calorimeter data for pine siding and pine studs at exposures of 25, 50, and 75 kW/$\hbox {m}^2$ was taken from https://materials.fsri.org [18] with replicate tests averaged. The resulting averaged curves were used as FDS inputs. Heat flux meter data from the website for the pine stud was used to obtain the room temperature specific heat and conductivity. Specific heat at higher temperatures was based on simultaneous thermal (STA) analysis data for pine studs also taken from the website. The wood was assumed to have a 10% moisture content (it was not measured prior to testing). The latent heat of the water mass was added to the specific heat as a triangular peak in specific heat between 90 and 110 °C. The heat of pyrolysis for the wood was set to 189 kJ/kg based on the STA data. The ignition temperature was set to 365 °C [33].

A simulation was also performed using pine kinetics from the Aalto_Woods FDS validation cases. Kinetics are available from the FDS GitHub repository at https://github.com/firemodels/fds/blob/master/Validation [22].

The NIST testing ignited the wood cribs by packing a region of the cribs with excelsior and igniting the excelsior. An excelsior only test with the same excelsior mass was performed. In the FDS model, one Lagrangian particle per cell was added over a volume similar to that seen in the NIST pre-test photographs. The particles were assigned the heat release rate of the NIST excelsior only test.

**Fig. 19:**
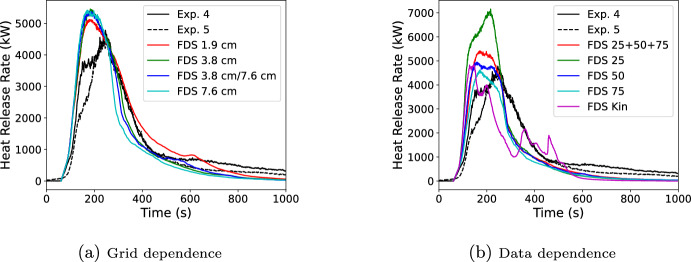
Measured vs. FDS predicted heat release rates for six wood pallets in an open corner. Left—All cone curves with varying grid resolution. Right—7.6 cm varied cone or kinetic data

Results of the FDS simulations are shown in Figure 19. The left figure contains results using all three cone curves and varying the grid resolution. The right figure contains results using the 7.6 cm grid while using either kinetic data or varying the cone data used. The first 60 s of the test were for background data collection. During the first 60 s of the fire (60–120 s test time), the heat release rate was primarily due to the Lagrangian particles representing the excelsior whose heat release peaks at 50 s post ignition. For the next 60 s all simulations closely track the initial spread of fire over the pallets.

In the grid study, little grid dependence was seen in both peak HRR and in the peak width, see Table 4. The 1.8 cm grid had a peak HRR that was 11% high and a peak width at half maximum that was 11% low. For the 3.8 cm, 7.6 cm, and mixed resolutions cases these were respectively 18% high and 7.2% low, 17% high and 21% low, and 16% high and 11% low. The peak HRR had little variance with the grid size and small changes are seen in the peak width.

**Table 4:** Grid (left) and data (right) study results for pallet HRR and peak width

Dataset	Peak HRR	Peak width	Dataset	Peak HRR	Peak width
	kW	s		kW	s
Test 4	4780	214	Test 4	4780	214
Test 5	4480	204	Test 5	4480	204
7.6 cm	5420	165	3.8 cm All	5450	194
3.8 cm	5450	194	3.8 cm 25	7160	159
1.9 cm	5122	187	3.8 cm 50	4940	174
Mixed	5390	186	3.8 cm 75	4630	164
			3.8 cm Kin	4850	137

In the data study, also shown in Table 4, more dependence was seen. The kinetic model does not capture the overall shape of the HRR curve. Its initial growth to peak was similar to the Spyro curves, but following the peak it shows a sharper decrease plus numerous other local peaks in HRR that are not present in either the data or the Spyro predictions. For errors in the peak HRR, the simulated HRR using data from all cone fluxes was 17% high compared to the experiment; the 25,50, and 75 kW/$\hbox {m}^2$ only curves are respectively 54%, 6.7%, and 0.0% high; and the kinetic curve was 4.8% high. Peak widths are all low compared to the experiment and in the respective order are 21%, 24%, 17%, 22%, and 34%. Overall the kinetic performance was not as good as the Spyro approach. It should be recognized that neither data set were the exact same wood burned in the experiments and each dataset used a different pine wood. However, while that might be expected to have an impact on the peak HRR and peak width, the shape behavior was poor with the kinetic approach. The all cone data and 50 and 75 kW/$\hbox {m}^2$ only curves had similar peak heights but the overall curve shape for the all and 50 were better than the 75. There was advantage to using multiple cone curves when available rather than selecting one curve and hoping it provides the best scaling.

Given the relative simplicity of the scaling approach and the very complex geometry resulting from voxelizing a stack of wood pallets, Spyro had good performance. Deviations over the longer term behavior were in part due to the FDS model not collapsing the pallet stack over time as it burned away and in part to (for the Spyro method) not fully accounting for char oxidation.

### Room Corner Tests

**Fig. 20:**
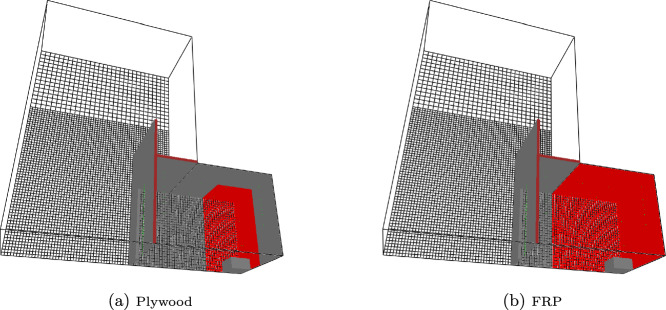
Room corner geometry for plywood (left) and FRP (right) lined room corner tests using Spyro. 8 cm grid shown. Wall lining shown as the red interior surfaces

**Fig. 21:**
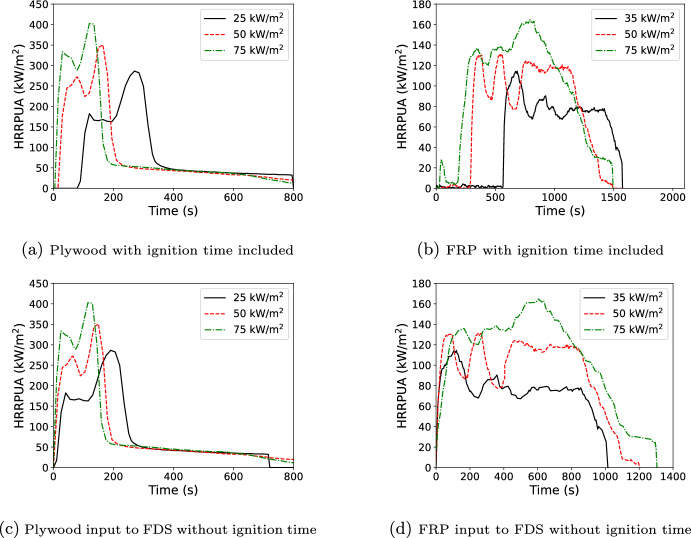
Cone data used in compartment fire simulations. The top row contains the full profile, and the bottom row is the input to FDS without the time to ignition. The left column is for plywood and the right column for FRP

This final validation test was a series of room corner tests [34] that took place in 1/4, 1/2, and full-scale NFPA 286 fire compartments [35]. The full scale compartment was 2.44 m wide, 3.66 m deep, and 2.44 m tall with the scaled compartments scaled the same for all dimensions. The full-scale had a 0.78 m wide by 2.1 m tall door. Door height was scaled with compartment dimensions. Door width was 0.55 and 0.4 m respectively for the 1/2 and 1/4 scale compartments. One corner opposite the door was lined with either 3.2 mm of a fiber reinforced, vinyl ester resin polymer (FRP) or 6.4 mm of plywood. Wall thickness was not scaled. The corner was lined from floor to ceiling plus the ceiling out to a distance of 1/2 of the short wall for plywood or the full short wall distance for the FRP. (e.g., 1.22 m or 2.44 m for the full-scale compartment). A propane burner (0.6 m by 0.6 m by 0.3 m for the full-scale and scaled with compartment dimensions) was placed in the corner and used to deliver a 40, 160, or 1430 kW fire. The full-scale was modeled at 8 cm (LR in plot legends) and 4 cm (HR in plot legends) grid resolution with the other compartments scaled proportionately. The mesh was extended out from the doorway by one compartment depth and above the doorway by one compartment height at the same grid resolution followed by one compartment height at a reduced grid resolution. Figure 20 shows the full-scale geometry for the plywood and FRP linings. Input files and cone data can be found in the FDS GitHub repository (https://github.com/firemodels/fds) and FDS experimental data repository (https://github.com/firemodels/exp). One example, the 1/4 scale plywood test, is provided as supplementary material. The plywood used cone exposures of 25, 50 and 75 kW/$\hbox {m}^2$, and the FRP used cone exposures of 35, 50, and 75 kW/$\hbox {m}^2$ (see Figure 21). Note that the plywood tested in the cone and the compartment were both 6.4 mm thickness; whereas, the FRP material in the cone was 6.4 mm but 3.2 mm in the compartment. The thickness scaling approach discussed in this work was used in the models of the FRP compartment.

Just based on the similarity of the curves and the 1D validation results, there was an expectation of better Spyro performance for the plywood. The FRP is a particularly challenging material to model with the Spyro approach. The FRP material is a composite with a gel coating which briefly ignites before the underlying material begins to char. During this process, local flame extinction occurs within 60 s of the gel coating ignition. After extinction of the gel coating, the underlying material requires several minutes of additional exposure to reach sustained ignition. This is particularly evident in the 75 kW/$\hbox {m}^{2}$ exposure shown in Fig. 21b where the initial burning of the gel coat registered in the calorimetry; although, the same behavior was observed visually at all three exposures. This material was modeled in the compartment fire using the data from the cone experiments starting after sustained ignition.

The burning behavior after ignition observed across exposure levels also differs from the assumed behavior in the Spyro model. The peak HRRPUA increases monotonically with cone exposure as expected. However, the burning duration also increases with exposure which leads to an increase in total heat released with higher exposure. This increase is the opposite trend observed in most materials in the database and the change in total energy released differs from the assumption in the Spyro approach. As a result, operation in the interpolation mode presented in this paper with multiple curves is expected to perform better than extrapolation from a single curve.

**Fig. 22:**
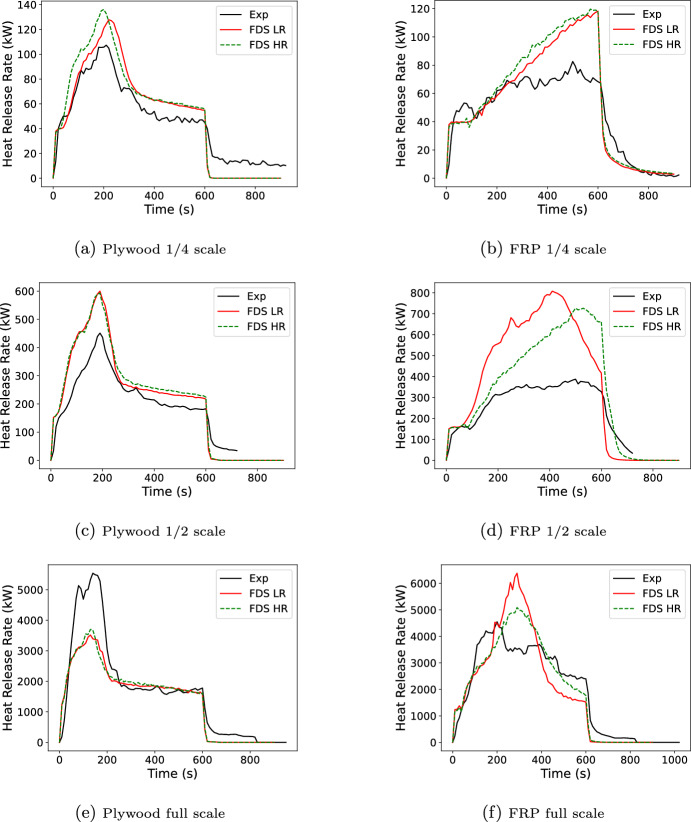
Comparison of FDS predicted burning rate for plywood (left) and FRP (right) lined room corner tests using Spyro for low (LR) and high (HR) resolution

**Fig. 23:**
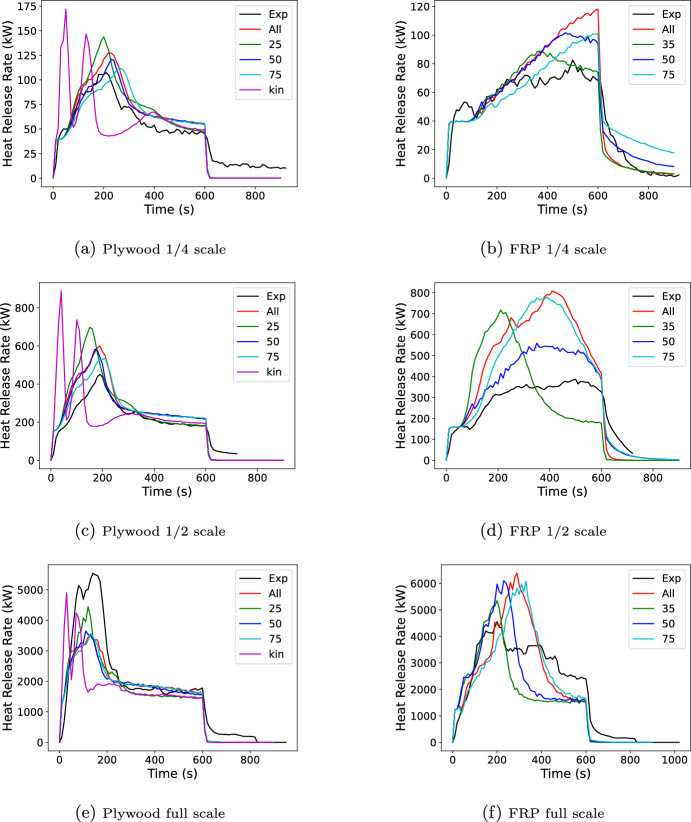
Comparison of FDS predicted burning rate for plywood (left) and FRP (right) lined room corner tests using Spyro with all cone data, each cone flux alone, or with kinetics (Plywood only)

Results are shown for the six tests in Figure 22 for the low and high resolution grids and Figure 23 for the low resolution grid with either all cone data, a single cone test, or kinetics (Aalto kinetics for the plywood case only).

For the plywood cases, the general shape of the experimental HRR curve (a rise to peak followed by a fall to a plateau) was predicted well by FDS. Little grid dependence was seen at 1/2 and full scale where the peak predictions were respectively 33% high and 37% low. At 1/4 scale the high resolution case was over predicting the peak by 27%, and the low resolution was over predicting by 19%. Peak widths were similar for all three scales with the high resolution peak for the 1/4 scale shifted in time compared to the lower resolution peak. For all scales and grid resolutions the peak time was similar to that in the data. For the post-peak plateau little grid dependence was seen. The average plateau from 400 to 600 s were over predicted by 25% for the 1/4 scale 21 to 27% for the 1/2 scale, and 2.8% for the full scale. The kinetic data with two large HRR peaks followed by a plateau showed poor performance in reproducing the general shape. The plateau portion was better predicted with results quite similar to the 25 kW/$\hbox {m}^2$ only results. Differences were seen when using one or three fluxes for the Spyro method. For this particular scenario, the three flux simulation was very similar overall to the results of the 50 kW/$\hbox {m}^2$ only simulation. At 1/4 and 1/2 scale the 75 kW/$\hbox {m}^2$ only simulation has the lowest peak error and at full scale it was the 25 kW/$\hbox {m}^2$ only simulation.

The FRP simulations did not reproduce the general shape of the experimental HRR curve as well as the plywood. The 1/4 and 1/2 scale simulations captured the slight delay before fire growth over the lining. The 1/4 scale simulation then showed a continuous rise to a peak 44% higher than the test peak for the lower resolution simulation. The test showed a brief rise to a plateau. Similar behavior was seen with 1/2 scale where the peak was 108% high. The full scale simulation more closely matched the general shape of a rise to a peak followed by a slow decrease until the ignition source was turned off; however, the post peak decay was more rapid and the peak was 40% high. The higher peaks observed in the model compared with the experiment are possibly due to the simplifying assumptions made in the cone calorimeter curves used in the model. Recall that the curve used in the model neglected the initial ignition and burn away of the gel coat, and the time delay during charring of the underlying material before the secondary ignition. As a result, the model over-predicts the burning rate compared with the experiment.

The FRP simulations showed more grid dependence than the plywood simulations. While the 1/4 scale showed little grid dependence, the 1/2 and full scale simulations showed grid dependence. In both cases the higher resolution case had a lower peak HRR which was closer to the measured data, with peak errors of 87% and 12% for the 1/2 and full scale, respectively. Much larger variations were seen when looking at the exposure data used. The plywood had differences at the peak but during the post-peak plateau had little difference with the cone data used. In contrast, the FRP simulations with different cone data had different results throughout the simulation. Different fluxes performed ”best” with the 35 kW/$\hbox {m}^{2}$ performing best at 1/4 scale, 50 at 1/2 scale and 75 at full scale. Since the FRP was not as well predicted, it cannot be definitively said that there was benefit in using one vs. three set of cone data for the FRP other than the challenge of being able to a priori pick the best single flux to use. However, these results highlight the difficulties that may arise when predicting fire growth with complex materials.

## Discussion

The FDS Spyro method of predicting burning rate was enhanced to utilize data from multiple cone calorimeter exposures and/or sample thicknesses. Utilizing cone data for predicting burning rate is attractive given modest material quantity needs for a cone calorimeter test, the availability of commercial testing labs, and the time and sample preparation required to perform a cone calorimeter test. Scaling approaches for flux and thickness were developed from dimensionless quantities for heat transfer and the energy balance for a quasi-steady burning surface. The method has been incorporated into the master branch of FDS.

The method requires determining the time dependent reference flux for a cone calorimeter test. This is not something that can be readily measured during a test without some interference with the test itself. A four variable space ($\Delta H_c$, $Y_s$, $\chi _r$, and HRRPUA) was modeled using FDS giving 1800 datapoints for ${\dot{q}}''_{flame}$ and $\Gamma$ that can be interpolated to define an empirical ${\dot{q}}''_{ref}$ for a cone calorimeter.

The developed method was used for five validation exercises:

1. A database of 141 materials with cone tests at multiple exposures was modeled using the method in solid phase only FDS simulations. Materials were divided into four categories: polymers, wood-based, mixtures, and other materials. When each cone exposure was predicted using the other available data, the first peak HRRPUA was predicted an unbiased with a 21% error. Polymers were predicted best overall (14% error), followed by wood (21%), other (22%) and mixtures (28%). The method performed best when the cone curves for material were highly self-similar, had a monotonic increase in peak burning rate with cone exposure, and lacked periods of extinction.
2. A subset of materials for the first exercise were tested at multiple thicknesses as well as multiple exposures. Applying thickness scaling to these materials had a first peak HRRPUA error of 37% with a bias of 1.08. This higher error and bias than for flux scaling was largely a result of the use of a higher proportion of mixtures and that the single wood material used was particularly challenging for the method. Polymers, mixtures, and other materials had similar error and bias to the flux scaling validation.
3. The burning rate of black PMMA was predicted for a 3D model of a cone calorimeter test and a model of a single burning item test. Simulations were performed with two detailed kinetics schemes and the Spyro method using three different cone data sets which included an inert dataset and a dataset at a different thickness. Predictions for the cone geometry were very similar to the measured data and closer than the kinetic approaches. The Spyro method was one-third low for the SBI test with little grid or data source dependence. The kinetic method was one-half low.
4. A stack of wood pallets in an open corner was predicted at two grid resolutions. The general timing of fire growth and decay was captured and little grid dependence was seen with this significantly more complicated geometry. Errors ranged from 11% to 17% high with peak width errors that ranged from 7.2% to 21% low. The long term fire behavior, dominated by char oxidation, was not well captured; however, the cone tests used in the model ceased data collection after flaming combustion ended.
5. Two sets of materials (plywood and an FRP) burned in 1/4, 1/2, and full-scale NFPA 286 compartments were modeled. Plywood predictions had little grid dependence and generally captured the trends in fire growth and decay; however, the peak HRR ranged from $\approx$30% high for the 1/2 scale to $\approx$30% low for the full scale test. Predictions for the FRP test showed more grid dependence and did not capture the general time dependent trends seen in the test data. This was consistent with the first exercise where mixtures were not as well predicted as polymers or wood-based materials.

Spyro was able to predict fire growth for multiple scales, multiple geometries, and for wood and polymer fuels to within 20 to 40% of experiments. For PMMA in a cone or SBI configuration, Spyro performance was better than that for detailed kinetics. Spyro has promise for an engineering approach to modeling fire growth given it can capture the approximate hazard of fuels while only requiring cone calorimeter data that can be obtained with relatively minor effort. For usage in practice it is recommended to first perform a 1D validation exercise for the cone data being used similar to the one done in Section 4.1. Thin materials and FR materials can be challenging for the Spyro method but such materials could be weeded out with a brief validation process.

It is noted that Spyro is not a full replacement for detailed kinetics. It is a method that relies on interpolation and extrapolation of cone data. As observed in the validation efforts, for some materials this process does not work well. It was also seen in validation that extrapolating to low fluxes and accounting for local extinction of burning was challenging for Spyro. Its main benefits are the relative ease and cost of obtaining input data, the grid independence of the method given current FDS heat flux predictions, and that once ignition occurs it decouples the material temperature from the burning rate.

## Supplementary Information

Supplementary information is provided in the form of an exemplar FDS input file for each category of simulation performed. The files are described below where the file name shown is the end portion of the name.

- *Supplementary file 2.fds*: An example FDS input file determining ${\dot{q}}''_{ref}$ for a material with a 30 MJ/kg heat of combustion, 5% soot yield, and 30% radiative fraction for multiple-flux validation (Section 2.2).
- *Supplementary file 7.fds*: An example FDS input file for multiple-flux validation (Section 4.1.1) for the HIPS polymer from the FAA database.
- *Supplementary file 6.fds* An example FDS input file for multiple-thickness validation (Section 4.1.2) for the HIPS polymer from the FAA database.
- *Supplementary file 13.fds*: An example FDS input file for modeling black PMMA in a cone calorimeter using the detailed kinetics scheme from DBI-LUND (Section 4.2.1).
- *Supplementary file 14.fds*: An example FDS input file for modeling black PMMA in a cone calorimeter using the Spyro method using cone data from DBI-LUND (Section 4.2.1).
- *Supplementary file 4.fds*: An example FDS input file for modeling black PMMA in an SBI apparatus using the detailed kinetics scheme from DBI-LUND (Section 4.2.1).
- *Supplementary file 5.fds*: An example FDS input file for modeling black PMMA in an SBI apparatus using the Spyro method using cone data from DBI-LUND (Section 4.2.1).
- *Supplementary file 3.fds*:An example FDS input file for modeling black PMMA in an SBI apparatus using the Spyro method using cone data and bulk property data from DBI-LUND (Section 4.2.1).
- *Supplementary file 11.fds*: An example FDS input file for modeling black a stack of wood pallets in an open corner using the Spyro method using cone data from FSRI (Section 4.3).
- *Supplementary file 12.fds*: An example FDS input file for modeling black a stack of wood pallets in an open corner using the Aalto woods FDS validation case kinetic data (Section 4.3).
- *Supplementary file 8.fds*: An example FDS input file for modeling a 1/4 scale room corner test of plywood using the Spyro method using cone data from JH (Section 4.4).
- *Supplementary file 10.fds*: An example FDS input file for modeling a 1/4 scale room corner test of FRP using the Spyro method using cone data from JH (Section 4.4).
- *Supplementary file 1.csv*: A csv file providing a listing of all materials used in Section 4.1. The file provides the test lab, the material name, the tested material thickness(es), and the tested fluxes.

## Electronic Supplementary Material

Below is the link to the electronic supplementary material.

Supplementary file1 (CSV 18 kb)

Supplementary file2 (FDS 96kb)

Supplementary file3 (FDS 50 kb)

Supplementary file4 (FDS 12kb)

Supplementary file5 (FDS 50 kb)

Supplementary file6 (FDS 23 kb)

Supplementary file7 (FDS 1 kb)

Supplementary file8 (FDS 16 kb)

Supplementary file9 (FDS 11 kb)

Supplementary file10 (FDS 23 kb)

Supplementary file11 (FDS 241 kb)

Supplementary file12 (FDS 188 kb)

Supplementary file13 (FDS 412 kb)

Supplementary file14 (FDS 451 kb)

## Data Availability

All experimental data in this paper are available at one or more of the locations in the list below. FDS experimental data repository: https://github.com/firemodels/exp. FSRI Materials and Products Database: https://materials.fsri.org. MaCFP condensed phase database: https://github.com/MaCFP/matl-db. MaCFP database: https://github.com/MaCFP/macfp-db.
